# Influence of Peptide Conjugation Sites on Lunatin–Alumina Nanoparticles: Implications for Membrane Interaction and Antimicrobial Activity

**DOI:** 10.3390/ph18070952

**Published:** 2025-06-24

**Authors:** Carolina Silva Ferreira, Lívia Mara Fontes Costa, Lúcio Otávio Nunes, Kelton Rodrigues de Souza, Giovanna Paula Araújo, Evgeniy S. Salnikov, Kelly Cristina Kato, Helen Rodrigues Martins, Adriano Monteiro de Castro Pimenta, Jarbas Magalhães Resende, Burkhard Bechinger, Rodrigo Moreira Verly

**Affiliations:** 1 Department of Chemistry, Federal University of the Jequitinhonha and Mucuri Valleys, Diamantina 39100-000, MG, Brazil; carolinasilvaferreirac@gmail.com (C.S.F.); livia.torres@ict.ufvjm.edu.br (L.M.F.C.); lucio.otavio@ufvjm.edu.br (L.O.N.); giovanna.araujo@ufvjm.edu.br (G.P.A.); 2 Institute of Science and Technology, Federal University of the Jequitinhonha and Mucuri Valleys, Diamantina 39100-000, MG, Brazil; 3 Institute de Chimie, Université de Strasbourg/CNRS, URM7177, 67000 Strasbourg, France; kelton.ufvjm@gmail.com (K.R.d.S.); e.salnikov@gmail.com (E.S.S.); bechinge@unistra.fr (B.B.); 4 Faculty of Pharmacy, Federal University of the Jequitinhonha and Mucuri Valleys, Diamantina 39100-000, MG, Brazil; kelly.kato@ufvjm.edu.br (K.C.K.); helen.rmartins@gmail.com (H.R.M.); 5 Department of Biochemistry and Immunology, Institute of Biological Sciences, Federal University of Minas Gerais, Belo Horizonte 31270-901, MG, Brazil; apimenta@gmail.com; 6 Segal Proteomics Centre, Lady Davis Institute for Medical Research, Jewish General Hospital, McGrill University, Montreal, QC H3T 1E2, Canada; 7 Department of Chemistry, Federal University of Minas Gerais, Belo Horizonte 31270-901, MG, Brazil; jarbasjmr@gmail.com

**Keywords:** nanobiomaterials, antimicrobial peptides, alumina nanoparticles, antibacterial activity, lunatin-1

## Abstract

**Background/Objectives:** The increasing prevalence of multidrug-resistant bacteria presents a major global health challenge, prompting a search for innovative antimicrobial strategies. This study aimed to develop and evaluate a novel nanobiostructure combining alumina nanoparticles (NPs) with the antimicrobial peptide lunatin-1 (Lun-1), forming peptide-functionalized nanofilaments. The main objective was to investigate how the site of peptide functionalization (C-terminal vs. N-terminal) affects membrane interactions and antibacterial activity. **Methods**: NP–peptide conjugates were synthesized via covalent bonding between lun-1 and alumina NP and characterized using transmission electron microscopy (TEM), X-ray diffraction (XRD), zeta potential analysis, dynamic light scattering (DLS), Fourier-transform infrared (FTIR), and solid-state ^13^C NMR. Antibacterial activities were assessed against different Gram-positive and Gram-negative strains. Biophysical analyses, including circular dichroism (CD), isothermal titration calorimetry (ITC), differential scanning calorimetry (DSC), and solid-state ^2^H NMR, were employed to evaluate peptide–membrane interactions in the presence of membrane-mimetic vesicles composed of POPC:POPG (3:1) and DMPC:DMPG (3:1). **Results**: Characterization confirmed the successful formation of NP–peptide nanofilaments. Functionalization at the N-terminal significantly influenced both antibacterial activity and peptide conformation compared to C-terminal attachment. Biophysical data demonstrated stronger membrane interaction and greater membrane disruption when lun-1 was conjugated at the N-terminal. **Conclusions**: The site of peptide conjugation plays a crucial role in modulating the biological and biophysical properties of NP–lunatin-1 conjugates. C-terminal attachment of lunatin-1 retains both membrane interaction and antibacterial efficacy, making it a promising strategy for the design of peptide-based nanotherapeutics targeting resistant pathogens.

## 1. Introduction

The emergence of multidrug-resistant bacteria, commonly known as “superbugs”, represents a critical and escalating global threat to public health. According to the World Health Organization (WHO), antimicrobial resistance (AMR) has reached an alarming proportion and is projected to surpass cancer as a leading cause of death within the next 25 years. The WHO has declared AMR as one of the top ten public health threats facing humanity [1]. Furthermore, data from the Global Antimicrobial Surveillance System (GLASS) reports a high prevalence of antibiotic resistance among 500,000 individuals with suspected bacterial infections across 22 countries. The most commonly identified resistant pathogens include *Escherichia coli*, *Klebsiella pneumoniae*, *Staphylococcus aureus*, *Streptococcus pneumoniae*, and *Salmonella* spp. [1].

In this context, there is an urgent need to develop alternative antimicrobial agents that are less likely to promote the emergence of bacterial resistance. Antimicrobial peptides (AMPs) have garnered considerable attention as promising alternatives, prompting extensive research into their synthesis and the elucidation of their molecular, biophysical, and biological properties, aiding a better understanding of how they act without promoting bacterial resistance. According to previously published studies, AMPs present a particularly compelling strategy to expand the therapeutic arsenal, especially in the context of treatment of resistant bacterial infections [2,3].

Although the mode of action of AMPs against pathogens is not yet fully understood, their interaction with the surface of bacterial cells is known to be a critical step. The interaction typically results in disruption or permeabilization of the phospholipid membrane, leading to cell death [2,3,4]. As a result, antimicrobial peptides exhibit potent antimicrobial activity with high selectivity toward pathogens, in contrast compared to many conventional antibiotics [5,6].

However, peptides are susceptible to rapid proteolytic degradation in both the gastrointestinal tract and bloodstream, which is one of the greatest obstacles in their development as therapeutic agents. An alternative strategy for enhancing their pharmacokinetic stability and extending their systemic half-life is the functionalization of the peptide with nanoparticles [7].

Recently, there has been growing interest in exploring the properties of nanoparticles for biological applications, particularly as alternative carriers for therapeutic peptides, proteins, and antigens [6,8,9]. Nanoparticles offer several key advantages in the context of antibiotic delivery: (i) ultra-small size, which is ideal for targeting intracellular bacteria and performing antimicrobial actions [10,11]; (ii) protect the drug from degradation or resistance mechanisms, as nanoparticle carriers can help enhance the serum levels of antibiotics [6]; and (iii) precision and safety, by enabling the targeted delivery of antibiotics to the site of infection while minimizing systemic side effects [12].

This work describes the synthesis, characterization, and antimicrobial potential of alumina-based nanobiostructures that are covalently bound to the C- or N-terminal portion of the antimicrobial peptide lunatin-1 (Lun-1). Lunatin-1 is a peptide isolated from the venom of the scorpion species *Hadruroides lunatus*, comprising 13 amino acid residues [13]. It was selected due its short primary structure (FIGGLLKTLTSFF–NH_2_) and its antimicrobial activity, exhibiting minimum inhibitory concentration (MIC) values ranging from 1.49 to 5.95 μmol·L^−1^ against Gram-positive *Streptococcus agalactiae*, *S. bovis*, *S. uberis*, and *Staphylococcus aureus* strains, and MIC values of 23.8 and 47.6 μmol·L^−1^ against Gram-negative *Escherichia coli* and *Pseudomonas fluorescens* strains, respectively [13]. On the other hand, alumina nanoparticles (NPs) were chosen for their bio-inertness and outstanding mechanical properties, such as high hardness, corrosion resistance, and durability against physical wear [14,15,16,17]. These nanoparticles have been extensively utilized in drug delivery systems [14,16], as well in biomedical applications such as dental implants and bone prostheses [18,19].

This study focuses on the synthesis of peptide-bound alumina nanoparticles using two derivative forms of lunatin-1, allowing functionalization at either the N-terminal (Lun-E_N_) or the C-terminal (Lun-E_C_) region. These peptide analogues incorporate three alanine (A) residues as spacers to promote the accessibility of the peptide chain for interaction with bacterial membranes. Additionally, the glutamic acid residue was included in each analog, serving as the covalent binding site for conjugation to functionalized alumina nanoparticles. Therefore, this study addresses how the site of functionalization at the C-terminal versus the N-terminal influences the membrane interaction profile and antibacterial activity of lunatin-1 when bound to alumina nanoparticles.

## 2. Results

### 2.1. Synthesis and Characterization

*Synthesis of peptides and nanostructures*: the peptide primary sequences, acronyms, and corresponding nanobiostructure representations are summarized in Table 1. Lunatin-1 (Lun-1) and its analogues (Lun-E_C_ and Lun-E_N_) were synthesized using solid-phase peptide synthesis (SPPS). The products were purified by reverse-phase high-performance liquid chromatography (HPLC) and characterized by mass spectrometry.

**Figure 1:**
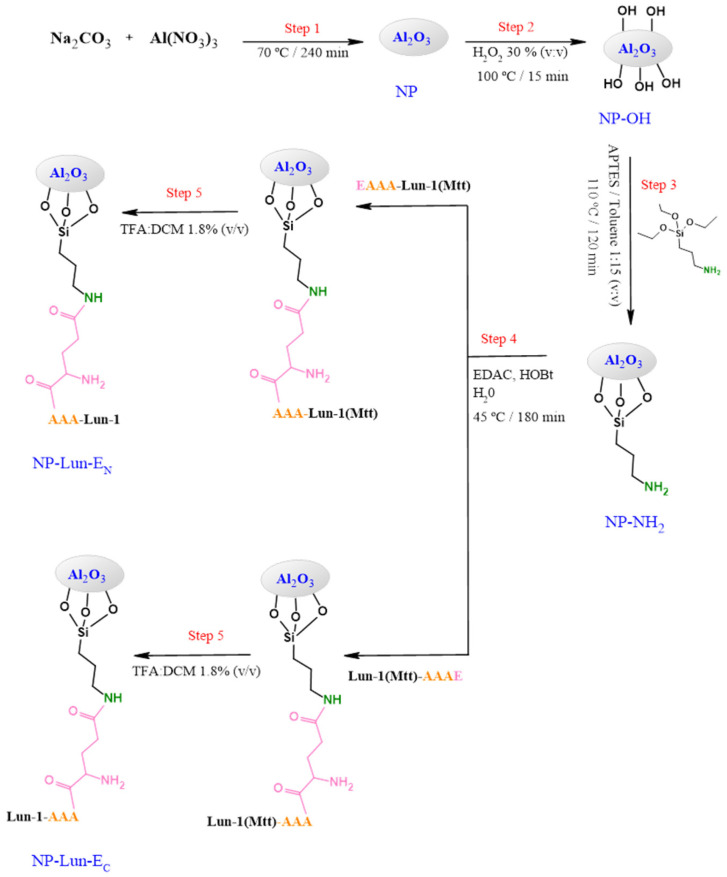
Synthetic pathway to obtain alumina nanobiostructures based on EAAA-Lun-1 and Lun-1- AAAE peptides.

The observed pseudo-molecular ions corresponding to Lun-1, Lun-E_C_, and Lun-E_N_ peptides were detected as shown in Figure S1. A glutamic acid residue was added to introduce a carboxyl group for activation with EDAC, enabling subsequent binding to the amine groups of the nanoparticle (NP), since the analogue peptides possess an amidated C-terminus. In addition, Lys-7, protected with the N-methyltrityl (Mtt) group, was incorporated to prevent side reactions (Figure S1d,e) and was removed after the cleavage step or the NP–peptide conjugation step. The stepwise synthesis of the NP–peptide conjugates is shown in Figure 1. Morphological and structural characterization of alumina nanoparticles and the functionalization steps were accessed using TEM, DRX, zeta-potential, FTIR, and ssNMR.

**Figure 2:**
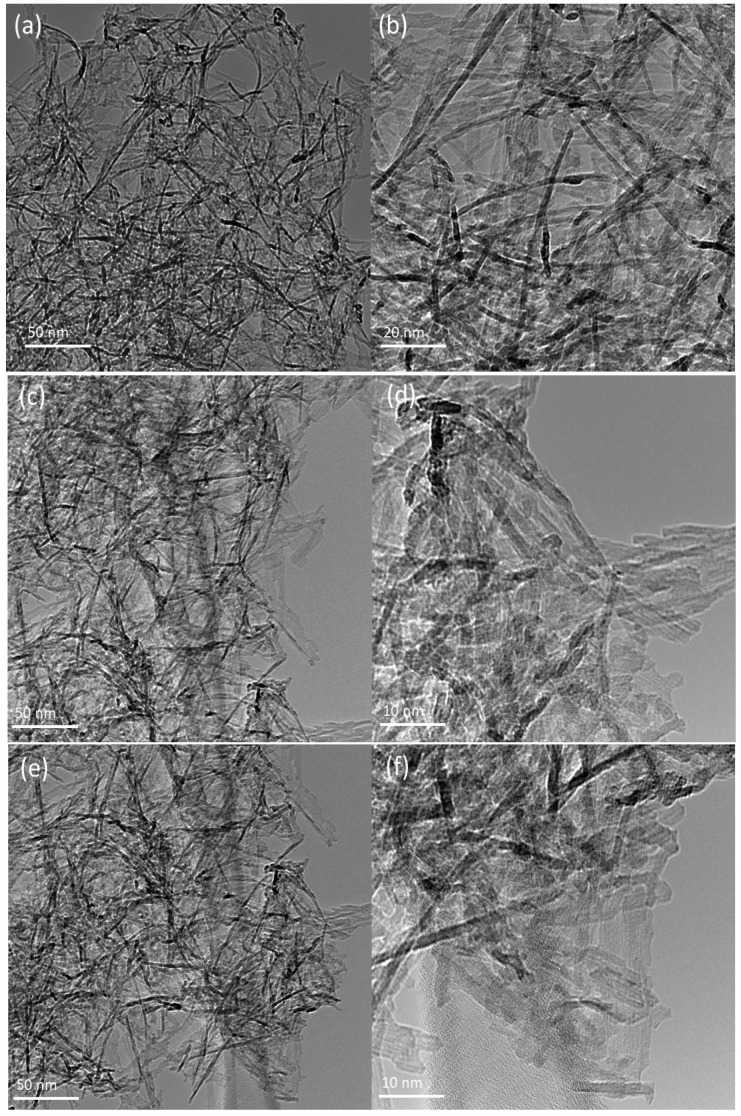
Transmission electron microscopy images of pure Al_2_O_3_ NP (**a**,**b**) and conjugated nanoparticles NP-Lun-E_N_ (**c**,**d**) and NP-Lun-E_C_ (**e**,**f**).

*Transmission Electron Microscopy and Powder X-ray Diffraction:* the morphologies of the NP, NP-Lun-E_N_, and NP-Lun-E_C_ nanoparticles were investigated using TEM. The images presented in Figure 2 were recorded at two different resolutions. The synthesized Al_2_O_3_ nanoparticles displayed a homogeneous morphology, structured in nanofilaments exhibiting a diameter within the range of 5 nm and an average length ranging from 20 to 100 nm. (Figure 2a,b). Notably, peptide functionalization does not induce significant changes in the fibrous nature of the nanostructures (Figure 2c–f). The crystal structures of Al_2_O₃ nanoparticles (NPs) and NP–peptide conjugates were investigated by X-ray diffraction (XRD) (Figure S2). The nanoparticles showed similar XRD pattern of the g-Al_2_O_3_ crystallites reported by Torres et al. (2018) [20].

*Zeta Potential and Hydrodynamic Diameter:* changes in the zeta potential and hydrodynamic diameter of the nanoparticles were monitored by dynamic light scattering (DLS) measurements after each synthesis step (Figure S3). Samples were dispersed in a 10 mM Tris-HCl buffer, pH 8.0, containing 100 mM NaCl. Non-functionalized alumina nanoparticles (NPs) exhibited a ζ-potential of −13.2 ± 0.9 mV. Similarly, the hydroxylated nanoparticles (NP-OH) showed a ζ-potential of −13.1 ± 0.5 mV, indicating no significant change when compared to the unmodified nanoparticles. In contrast, the amidation of the nanoparticles (NP-NH_2_) led to a significant shift in surface charge, yielding a positive ζ-potential of +19.9 ± 0.5 mV. For the nanoparticles functionalized with Lun-1, specifically NP-Lun-E_N_ and NP-Lun-E_C_, the ζ-potential values were +6.7 ± 0.9 mV and +5.8 ± 0.8 mV, respectively), regardless of whether the peptide was conjugated at the C-terminus or the N-terminus. Hydrodynamic diameter measurements (Figure S4) revealed larger values for NP and NP-OH (1850 nm and 3230 nm, respectively), suggesting limited colloidal stability. The functionalization with amino groups reduced the average size to approximately 1400 nm. A further decrease in hydrodynamic diameter was observed upon both peptide conjugations, with NP–peptide systems displaying the smallest sizes among the tested formulations. These findings suggest enhanced colloidal stability [21], as no visible aggregation or sedimentation was observed over 24 h.

**Figure 3:**
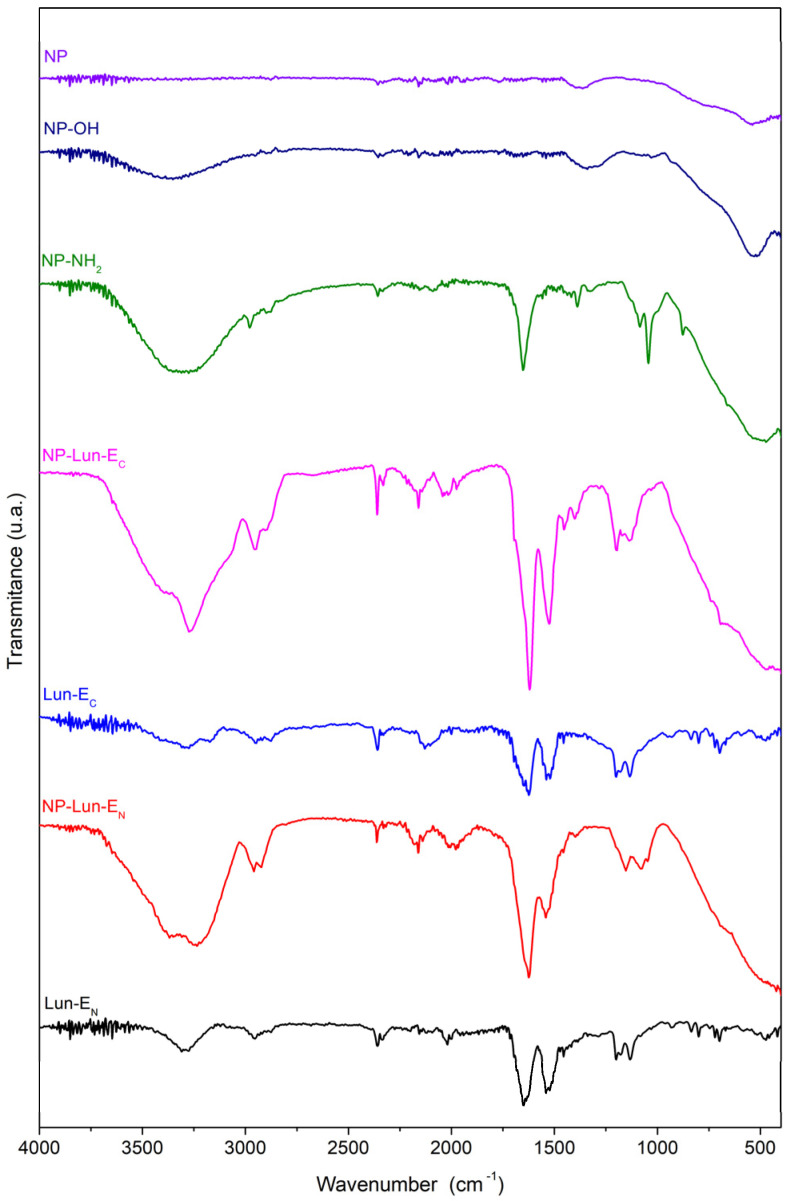
FTIR spectra of nanoparticles and peptide-functionalized systems: NP, NP-OH, NP-NH_2_, NP-Lun-E_C_, Lun-E_C,_ NP-Lun-E_N_, and Lun-E_N_. The spectra highlight the chemical modifications at each functionalization stage and the presence of characteristic vibrational bands associated with peptide conjugation.

*Fourier transform infrared spectroscopy:* the FTIR-ATR technique was employed to characterize the functional groups and confirm the successful completion of every step in the syntheses of the nanobiostructures. Samples of free peptides (Lun-E_N_ and Lun-E_C_) and nanoparticles (NP, NP-OH, NP-NH_2_, NP-Lun-E_N_, and NP-Lun-E_C_) were analyzed in their solid state using an ATR device (Figure 3). Distinct characteristic vibrational bands corresponding to the functional groups present in each nanoparticle were observed. The spectrum of NP shows a vibrational band within the range of 1110–800 cm^−1^, which was seen to be consistently present across all spectra containing nanoparticles and characteristic of the Al-O-Al bond. The FTIR spectrum of NP-OH exhibits a broad hydroxyl stretching band between 3690–3000 cm^−1^, while the spectrum of NP-NH_2_ reveals a distinct high-intensity stretch in the range of 3250–3500 cm^−1^, accompanied by two smaller bands below 3000 cm^−1^, confirming the presence of the primary amine and methylene groups, respectively. Additionally, strong signals are identified in the regions of 1350–1000 cm^−1^ and 1650–1580 cm^−1^, characteristic of amine C-N bond stretching and N-H bend of amines, respectively. The spectra of free peptides and peptides bound to nanoparticles present a prominent band at 1650 cm^−1^, corresponding to the axial deformation vibrations of the carbonyl group (C=O), while the band at 1535 cm^−1^ is attributed to the symmetric in-plane angular deformation vibrations of the N-H bond. Axial deformation of the C-N bond is observed, along with vibrations near 3300 cm^−1^ corresponding to the axial deformation of N-H and C-H bonds. Additionally, the spectra display bands associated with C-H stretching vibrations of phenylalanine aromatic rings, with significant features in the range of 900–690 cm^−1^ (harmonics between 2000 and 2300 cm^−1^) and 1600–1475 cm^−1^, corresponding to C=C stretching vibrations of aromatic rings.

**Figure 4:**
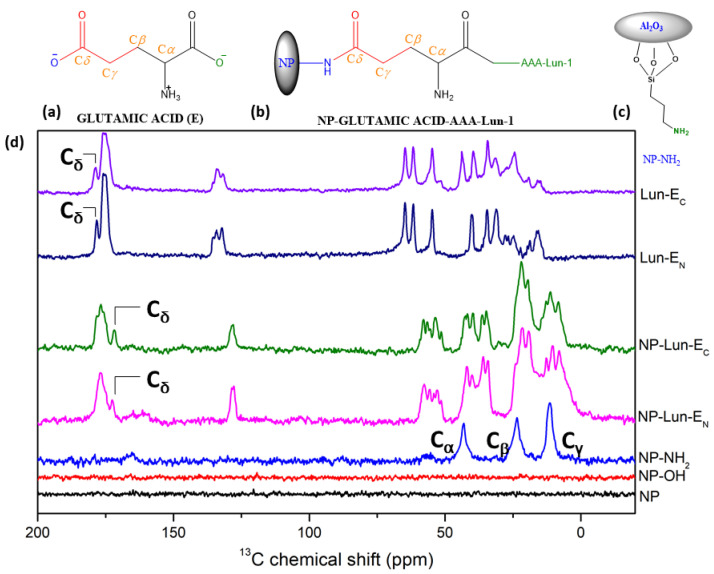
Chemical structure of glutamic acid (**a**); NP-Lun-E_N_ (**b**) highlighting the binding regions of the nanoparticle. Structure of NP-NH_2_ showing the Ca, Cb, and Cg. Chemical structure of NP-NH_2_ showing the Ca, Cb, and Cg (**c**). ^13^C ssNMR spectra of NP, NP-OH, NP-NH_2_, NP-Lun-E_N_, NP-Lun-E_C_, Lun-E_N_, and Lun-E_C_ (**d**).

*Solid-State Nuclear Magnetic Resonance spectroscopy:* ^13^C ssNMR spectroscopy was employed to characterize every stage of nanoparticle functionalization, providing comprehensive information on the organic groups covalently bound to the Al_2_O_3_ nanoparticle. For comparative analysis, spectra of the free peptide were also acquired, and the results are presented in Figure 4. As expected, no ^13^C signals were observed for either NP or NP-OH (Figure 4d, black and red line). However, for NP-NH_2_ (Figure 4d, blue line), intense signals were observed at **11.3 ppm, 23.4 ppm, and 43.1 ppm**, which correspond to the α, β, and γ methylene groups of the nanoparticles functionalized with APTES (Figure 4c). The ^13^C NMR spectra of the free peptides Lun-E_N_ (Figure 4d, dark blue line) and Lun-E_C_ (Figure 4d, purple line) were similar, with intense signals observed in the **175–185 ppm** range. In the spectra of nanoparticles conjugated with peptides (Figure 4d, green and magenta lines), signals resembling those of the free peptides were observed. The signals near to **183 ppm** for both free peptides are assigned to the carbonyl side chain C_δ_ of glutamic acid, as represented in Figure 4a,b. In both NP-Lun-E_N_ and NP-Lun-E_C_, this signal appears at a lower chemical shift (**176.8 ppm**), indicating a change in the chemical environment from carboxyl to amide group, consistent with covalent binding between the carbonyl side chain and NP-NH_2_.

### 2.2. Biological Assays

*Antibacterial assay:* The Lun-1 peptide and the nanobiostructure NP-Lun-E_C_ demonstrated activity against all species evaluated (Table 2). In contrast, NP-Lun-E_N_ did not exhibit activity against *Acinetobacter* and *Pseudomonas aeruginosa*. Among the two nanobiostructures, NP-Lun-E_C_ displayed superior efficacy, as reflected by its lower minimum inhibitory concentration (MIC) values compared to NP-Lun-E_N_. Notably, when comparing the MIC values of NP-Lun-E_C_ with those of the free Lun-1 peptide, the peptide-functionalized nanoparticle exhibited comparable MIC values across all tested bacterial strains, indicating that C-terminal functionalization does not significantly impact antibacterial activity.

**Figure 5:**
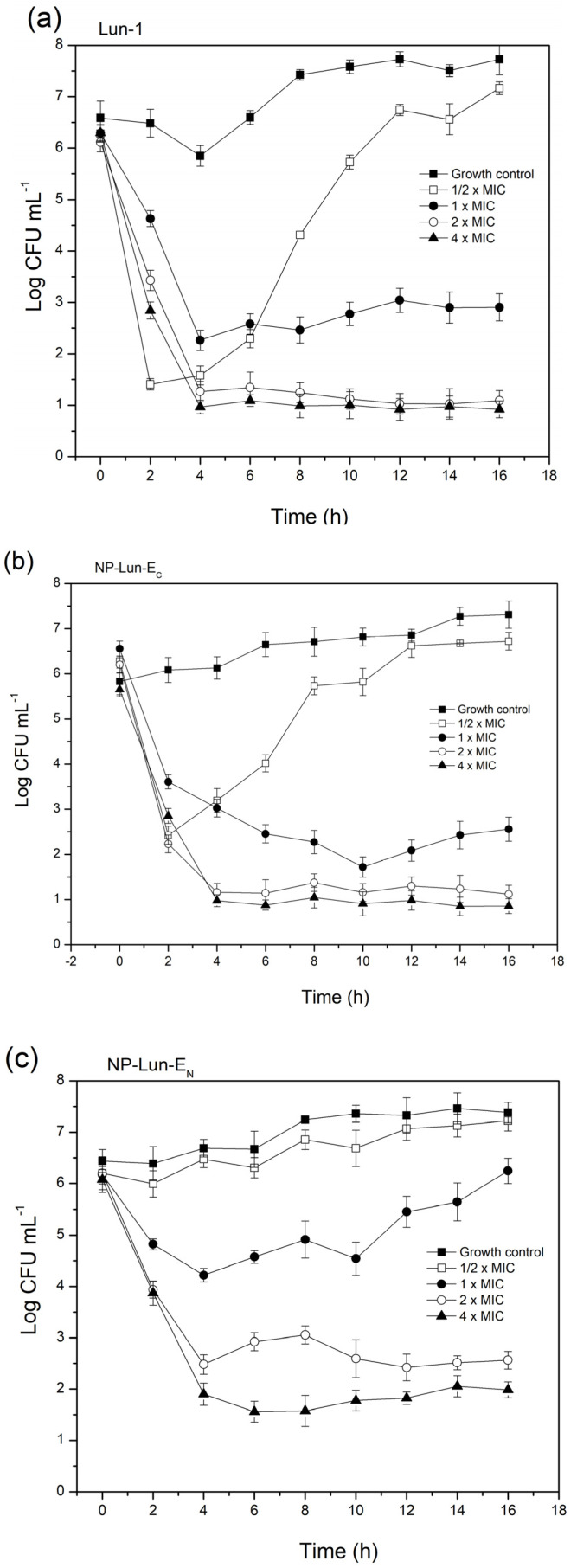
Bacterial cell death kinetics for *Staphylococcus aureus* cultured in MH broth at 10^5^ CFU·mL^−1^ and exposed to the free Lun-1 peptide (**a**) and nanobiostructures NP-Lun-E_C_ (**b**) and NP-Lun-E_N_ (**c**) at 0.5×, 1×, 2×, and 4× the MIC. Samples were collected at time intervals of 0, 2, 4, 6, 8, 10, and 12 h.

*Bacterial cell death kinetics*: The results obtained for the *Staphylococcus aureus* bacterial cell viability over time are presented in Figure 5. For Lun-1 (Figure 5a), a sharp drop in viable bacteria was observed within the first four hours at peptide concentrations of 1 × MIC, 2 × MIC, and 4 × MIC, with no signs of subsequent regrowth. At ½ × MIC, an initial decline in viability occurred during the first two hours, followed by a plateau and then a significant increase, indicating the resumption of exponential growth by the surviving cells. A similar pattern was observed for NP-Lun-E_C_ (Figure 5b), where regrowth occurred at ½ × MIC, while concentrations ≥ 1 × MIC maintained a sustained reduction in viable cells. In contrast, NP-Lun-E_N_ (Figure 5c) showed a curve at ½ × MIC similar to that of the control, suggesting minimal antibacterial activity. At 1 × MIC, there was a transient reduction in viability during the first four hours, followed by regrowth, implying that some bacterial cells remained viable and were able to proliferate. Sustained suppression of bacterial viability was only reached at 2 × MIC and 4 × MIC. These observations suggest that under the experimental conditions, the apparent MIC may be underestimated, potentially due to peptide sequestration by released intracellular components, and highlight the importance of interpreting MIC values in the context of dynamic bacterial survival and regrowth.

### 2.3. Evaluation of Peptide–Membrane Interactions

**Figure 6:**
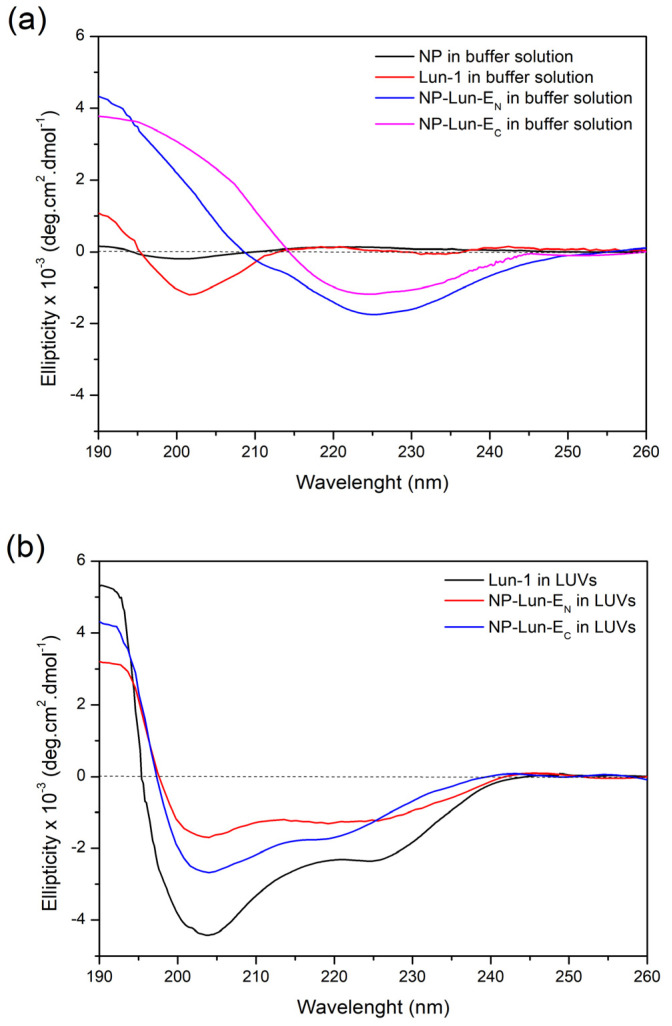
Circular dichroism (CD) spectra of NP (0.155 mg·mL^−1^), NP-Lun-E_N_ (0.172 mg·mL^−1^), NP-Lun-E_C_ (0.181 mg·mL^−1^), and Lun-1, all at a peptide concentration of 25 µM. (**a**) Spectra acquired in 10 mM Tris-HCl buffer containing 100 mM NaCl, pH 8.5. (**b**) Spectra recorded in the presence of 200 µM POPC:POPG (3:1) LUVs suspended in the same buffer.

*Circular Dichroism:* circular dichroism experiments were conducted to evaluate the conformational behavior of free peptides and nanoparticle–peptide conjugates in the buffer solution (Figure 6a) or in the presence of POPC:POPG (3:1, mol:mol) LUVs (Figure 6b).

Free Lun-1 peptide showed no preferential conformation in buffer solution, as indicated by the minimum, near to 200 nm (spectra in red). Interestingly, when bound to the nanoparticles (NP-Lun-E_C_ and NP-Lun-E_N_), the peptides presented a minimum around 220 nm, indicating structural ordering even in aqueous solution. The CD deconvolution reveals β-sheet percentages of 45% and 40% and a-helical percentages of 35% and 30% for NP-Lun-E_N_ and NP-Lun-E_C_, respectively. In the presence of anionic LUVs, free peptide and NP–peptide conjugates presented a typical spectral profile of α-helical conformation, characterized by a maximum at 192 nm and two minima near to 210 and 222 nm. The α-helix content of Lun-1 (Figure 6b spectra in black) was 70%, whereas NP-Lun-E_C_ (Figure 6b spectra in blue) and NP-Lun-E_N_ (Figure 6b spectra in red) exhibited helical content of 55% and 40%, respectively.

**Figure 7:**
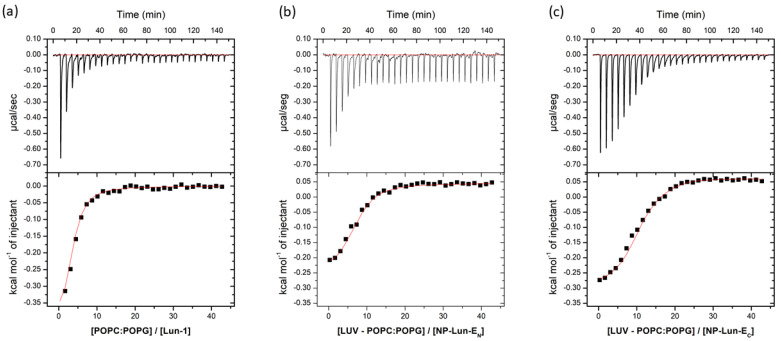
Heat flow isotherms for each injection as a function of time (upper section of each panel) and isotherms obtained by integrating the heat flow during the calorimetric titration of each sample solution (50 µM peptide concentration) with 20 mM LUVs composed of POPC:POPG (3:1, mol:mol) (lower section of each panel): (**a**) Lun-1; (**b**) NP-Lun-E_N_; (**c**) NP-Lun-E_C_. Samples and LUVs were suspended in 10 mM tris-HCl buffer at pH 8.5.

*Isothermal Titration Calorimetry:* the thermodynamic parameters of peptide–membrane interaction were obtained through ITC measurements, titrating POPC:POPG (3:1, mol:mol) LUVs into free peptide or NP–peptide conjugates at 50 µM peptide concentrations (Figure 7). All samples were suspended in 10 mM Tris-HCl, 100 mM NaCl, pH 8.5; the data were corrected by subtracting the heat flow from the titration of LUVs in buffer from the data obtained in every experiment. All systems revealed negative heat, indicative of predominantly exothermic interactions.

The thermodynamic parameters *n, K*, Δ*G^0^*, Δ*S^0^*, and Δ*H^0^*, obtained for the ITC experiments, are presented in Table 3. For the free peptide, the membrane interaction is predominantly exothermic (Δ*H*⁰ = –450 cal·mol^−1^) and spontaneous (Δ*G*⁰ = –5814 cal·mol^−1^), with a binding constant (*K*) of 3.0 × 10^4^ L·mol^−1^. When compared to the NP-Lun-E_C_ nanostructure, the binding constant remained within the same order of magnitude (4.1 × 10^4^ L·mol^−1^), while for NP-Lun-E_N_, the affinity constant is ten times lower (5.1 × 103 L·mol^−1^). Furthermore, NP-Lun-E_C_ exhibited higher thermodynamic values compared to both NP-Lun-E_N_, indicating that the N-terminal portion is important for peptide–membrane interactions.

**Figure 8:**
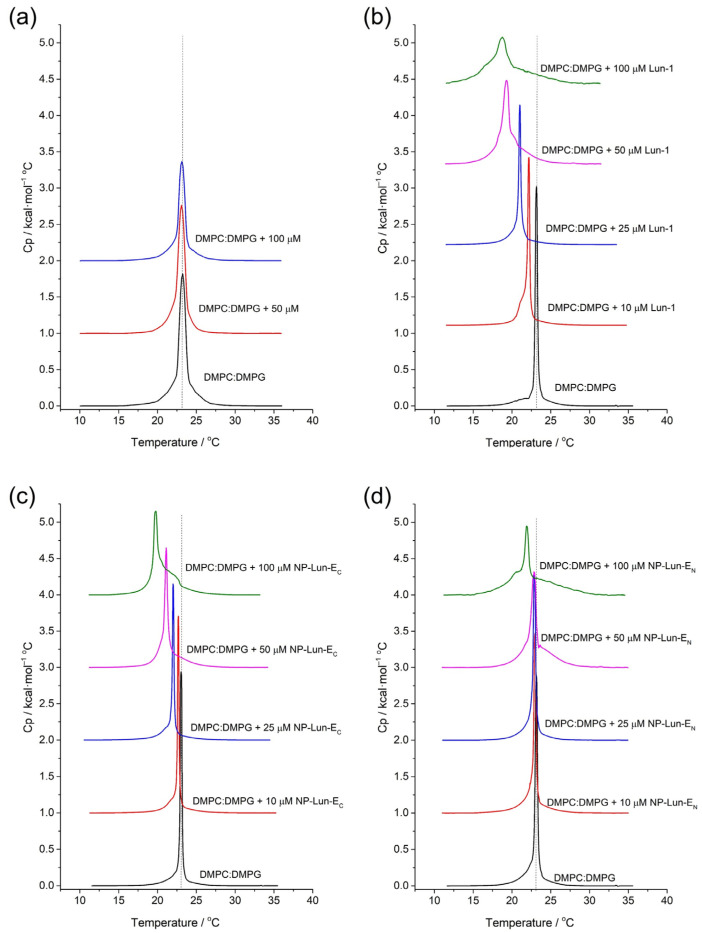
DSC curves for DMPC:DMPG (3:1, mol:mol) model membranes, without and with the addition, at varying concentrations: (**a**) NP; (**b**) Lun-1; (**c**) NP-Lun-E_N_; (**d**) NP-Lun-E_C_. The molar ratios for each curve are indicated on the respective graphs.

*Differential Scanning Calorimetry:* the effect of peptide–membrane interactions in the thermotropic profile of DMPC:DMPG (3:1, mol:mol) LUVs was investigated by Differential Scanning Calorimetry (DSC) (Figure 8). The thermotropic behavior of phospholipid vesicles was investigated in 10 mM Tris buffer (pH 7.5) and in the presence of NP, Lun-1, NP-Lun-E_N_, and NP-Lun-E_C_. The phase transition temperature (*T_m_*) and enthalpy of transition (D*_trans_H*) for all systems are presented in Table 4. The thermograms of pure DMPC:DMPG (3:1, mol:mol) LUVs shows a main peak corresponding to the *T_m_* of 23 °C and D*_trans_H* of 24 kJ·mol^−1^. The addition of NP does not appear to disturb the phospholipid membrane, as the endothermic peak remains unchanged at the same temperature in both NP concentration tested (Figure 8a). However, changes in the thermotropic profile of LUVs are evident with addition of the Lun-1 peptide (Figure 8b).

Decreased *T_m_* and broadening of the main peak occurs with increasing peptide concentrations. For the highest peptide concentration, the *T_m_* reaches 18.7 °C and the enthalpy of transition is 16.2 kJ·mol^−1^. Changes in the thermotropic profile are also observed for the NP–peptide samples (Figure 8c,d). Nevertheless, NP-Lun-E_N_ (Figure 8c) induced significant changes in both *T_m_* and D*_trans_H* only at the highest peptide concentration (100 µM), whereas NP-Lun-E_C_ caused noticeable disturbance in these thermodynamic parameters already at a 25 µM peptide concentration (Figure 8d), indicating a stronger interaction with the membrane.

**Figure 9:**
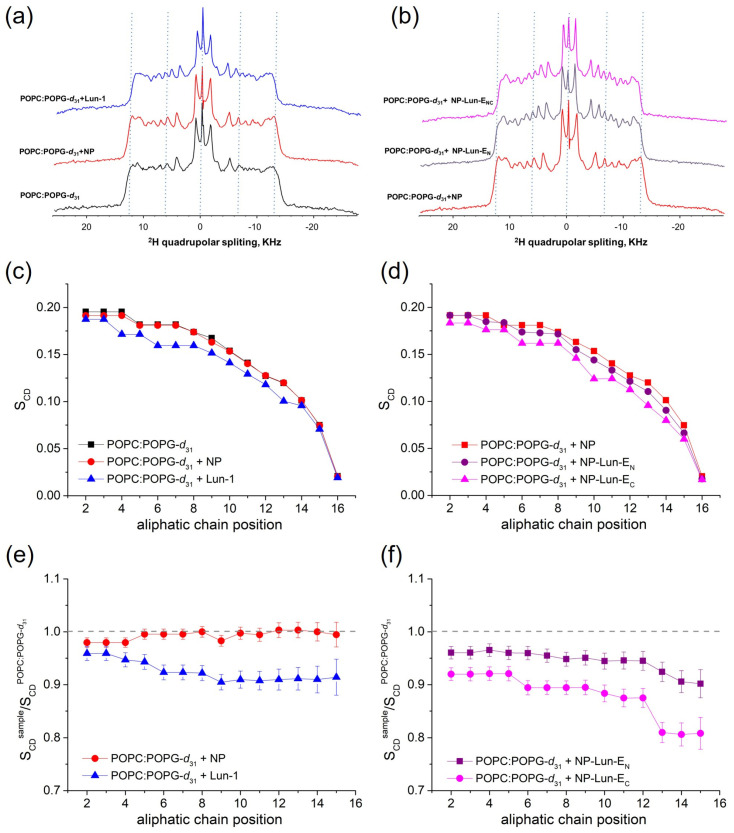
Solid-state ^2^H NMR spectra of lipids deuterated along the palmitoyl chain (**a**,**b**), order parameters (**c**,**d**), and relative order parameter profiles for POPC:POPG-*d_31_* (3:1, mol:mol) (**e**,**f**), in the absence and presence of NP (0.155 mg·mL^−1^), Lun-1, NP-Lun-E_N_ (2.782 mg·mL^−1^), and NP-Lun-E_C_ (2.888 mg·mL^−1^). All peptide-containing samples include 2 mol% peptide and were studied at pH 7.0. The experiments were conducted at 300 K. Dashed lines in panels A, B, E, and F are included as visual guides.

*Evaluation of phospholipid bilayer ordering by solid phase NMR:* the effect of the Lun-1 peptide and NP–peptides on lipid bilayer organization was evaluated by ^2^H solid-state NMR spectroscopy, which reveals the quadrupolar splitting of the deuterated palmitoyl chain of deuterated POPG-*d_31_*, as shown in Figure 9a,b. The ^2^H quadrupolar coupling values (Figure 9a,b) obtained for the methylene groups of POPC:POPG-*d_31_* (3:1, mol/mol) were converted into *S_CD_* order parameters (Figure 9c,d) and relative order parameters (Figure 9e,f) were subsequently calculated as the ratio of the *S_CD_* values of each sample to those of pure vesicles.

The spectrum of LUVs with nanoparticles (NPs) displayed similar order parameters across all methylene groups of the lipid chain when compared to those of pure LUVs (Figure 9c), resulting in relative order parameters close to 1 (Figure 9e). In contrast, the addition of Lun-1 reduced the quadrupolar couplings, leading to a significant decrease in order parameters, particularly from the carbon at position 3 on (Figure 9c). Comparing pure NP and the conjugated forms, NP-Lun-E_C_ notably induced greater changes in the lipid relative order parameters than NP-Lun-E_N_.

**Figure 10:**
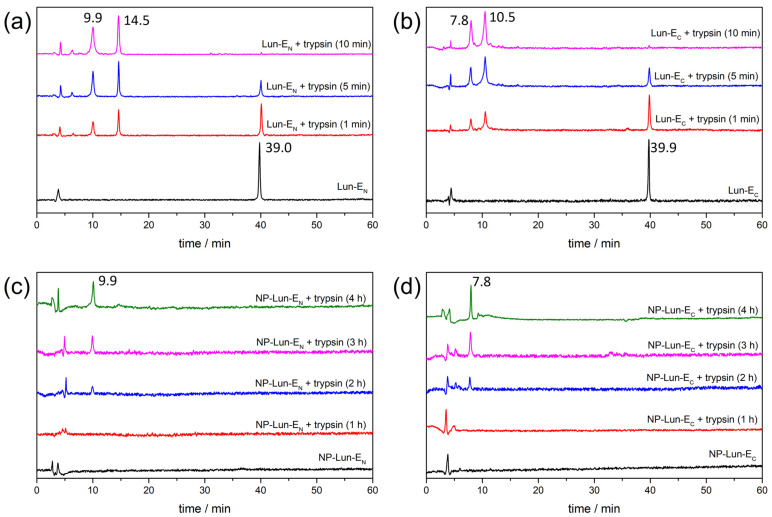
Proteolytic degradation profiles analyzed by HPLC of free and nanoparticle-conjugated lunatin-1 peptides in the absence and in the presence of trypsin. (**a**) Lun-E_N_, (**b**) Lun-E_C_, (**c**) NP-Lun-E_N_, and (**d**) NP-Lun-E_C_ samples were incubated with trypsin at different times.

*Proteolytic degradation studies*: proteolysis assays were conducted to evaluate the susceptibility of different conjugated and unconjugated forms of the lunatin-1 peptide to enzymatic degradation by trypsin over time (Figure 10). Chromatographic analyses by HPLC, performed after incubation at different time, reveal distinct degradation kinetics among the evaluated samples. Chromatograms of the free peptides Lun-E_N_ and Lun-E_C_ (Figure 10a,b) in the absence of enzymes reveal a main peak corresponding to the purified peptides, with retention times (Rt) of 39.0 min and 39.9 min, respectively. Complete degradation of both peptides occurs after 10 min in the presence of trypsin, as no significant signals are observed at 39.0 min or 39.9 min. The peaks with Rt of 9.9 min and 14.5 min in the chromatograms of Lun-E_N_, and Rt of 7.8 min and 10.5 min in those of Lun-E_C_, were collected and characterized by mass spectrometry, confirming the predicted cleavage fragments of each peptide, as presented in Table S2. In contrast, the nanoparticle–peptide conjugated NP-Lun-E_N_ and NP-Lun-E_C_ exhibit increased stability in the presence of trypsin (Figure 10c,d), with slower degradation over time. The main peak was not observed for either NP-Lun-E_N_ or NP-Lun-E_C_ in the absence of enzymes, since NP-conjugated suspensions were previously centrifugated and filtrated. A progressive degradation profile is observed only after 2 h in the presence of trypsin, with the appearance of peaks at Rt of 9.9 min for NP-Lun-E_N_ and Rt of 7.8 min for NP-Lun-E_C_. These peaks reveal the cleavage fragments from the peptide chains covalently bound to their respective NP–peptide conjugates.

## 3. Discussion

In this study, we characterize the morphologies, antimicrobial activities, and membrane interactions of alumina nanostructures functionalized with bioactive peptide lunatin-1. Transmission electron microscopy confirmed the formation of nanofilaments, which differ from the spherical alumina nanoparticles typically observed [22,23]. Although data on the benefits of nanofilament structures are still limited, this morphology may confer advantages in biomedical applications, such as bone prosthesis applications. Specifically, the nanofilament morphology appears to enhance osteoblast function, which is essential for the production of the bone’s organic matrix [24]. Surface charge changes throughout the functionalization process were monitored by zeta potential analysis, confirming the efficiency of each conjugation step. The non-functionalized nanoparticles (NPs) exhibited a ζ-potential of −13.2 ± 0.9 mV, which remained similar after hydroxylation (−13.1 ± 0.5 mV) [25]. While amidation shifted the charge to +19.9 ± 0.5 mV due to the presence of protonated amino groups [26], lunatin-1 conjugation reduced the ζ-potential as a consequence of the displacement of positively charged surface amino groups, reducing their contribution to the zeta potential [27,28].

Structural characterization through FTIR-ATR and ssNMR were employed to confirm the functionalization steps. FTIR spectra displayed characteristic vibrational bands for each functionalization stage [29,30]. Hydroxylation was confirmed by a broad O-H stretch (3690–3000 cm^−1^) [31], while amidation revealed N-H deformations (3250–3500 cm^−1^) of primary amine. Peptide conjugation was evident through characteristic peptide backbone vibrations in FTIR spectra, including C=O axial deformations (1650 cm^−1^) and N-H deformations (1535 cm^−1^) [32,33]. Aromatic ring vibrations confirmed phenylalanine residue was present in the nanoparticle structures. As expected for pure Al_2_O_3_, the ^13^C ssNMR MAS spectra of NP showed no carbon signals, while the functionalized samples exhibited distinct peaks attributed to the incorporated functional groups. The presence of APTES was supported by corresponding ^13^C signals at 11.3, 23.4, and 43.1 ppm. The ^13^C solid-state NMR spectra revealed distinct peptide structural features, indicating successful conjugation. Notably, the C_δ_ of the glutamic acid side chain in free Lun-E_C_ and Lun-E_N_ peptides appears at a higher ^13^C chemical shift (178 ppm), but shifts to a lower value (171 ppm) upon binding to the nanoparticles, confirming covalent attachment to the nanostructures.

Peptide loading on nanoparticles was quantified to assess their bioactivity. NP-Lun-E_N_ and NP-Lun-E_C_ exhibited similar functionalization degrees of 0.1438 and 0.1385 mmol·g^−1^, respectively, which are significantly higher than that of BP100–alumina nanoparticles (0.0455 mmol·g^−1^), obtained using the similar synthetic route [20]. The higher degree of functionalization may result from selective covalent bonding promoted by the synthetic route, which involved the use of protected lysine during NP–peptide conjugation (step 4, Figure 1). The antimicrobial assay (Table 2) demonstrated that the NP–peptide conjugates displayed antimicrobial activity, in contrast to the pure alumina nanoparticles, which exhibited no activity. Therefore, the antimicrobial efficacy of NP-Lun-E_N_ and NP-Lun-E_C_ against bacterial strains can be associated with the peptide molecule. Overall, NP-Lun-EC exhibited antimicrobial potential and MIC values comparable to those of the free peptide. In contrast, NP-Lun-E_N_ exhibited reduced efficacy compared to both Lun-1 and NP-Lun-E_C_. To further investigate this effect, bacterial cell death kinetics were evaluated using *Staphylococcus aureus*, given the pronounced difference in MIC values between the two peptide–nanoparticles in this bacterial strain. All tested systems exhibited dose-dependent killing. Lun-1 induced bacterial death within 2–4 h, which is consistent with the rapid action typically observed for membrane-targeting antimicrobial peptides [34,35]. NP-Lun-E_C_ presented a similar kinetic profile, supporting the conclusion that C-terminal functionalization preserves peptide bioactivity. In contrast, NP-Lun-E_N_ showed delayed and diminished bactericidal action, suggesting that N-terminal conjugation impairs membrane interaction and, consequently, antimicrobial efficiency. These findings highlight the fact that the terminal modifications of peptides play a crucial role in modulating their membrane interactions and biological activities. Kuzmin et al. (2017) showed that changes to the C-terminal region enhance lipid bilayer disruption, indicated by deuterium NMR data reflecting deeper membrane interaction [36]. Likewise, Li et al. (2016) demonstrated that modifications at both peptide termini influence their conformation and orientation within model membranes, significantly affecting their insertion depth and biological effectiveness [4].

Therefore, peptide–membrane interaction studies were performed with lipid mimetic systems (POPC:POPG and DMPC:DMPG, 3:1, mol:mol) using different techniques including ITC, CD, DSC, and ^2^H solid-state NMR. ITC confirmed spontaneous interaction (negative Gibbs free energy) [37], driven primarily by entropic contributions for all species investigated, i.e., Lun-1 and both NP–peptides. Nevertheless, NP-Lun-E_C_ exhibited stronger binding affinity than NP-Lun-E_N_, confirming a key role for the N-terminal region in peptide–membrane interaction. CD spectra indicated that NP-Lun-E_C_ adopted a α-helix conformation (helical content of 55%) compared to NP-Lun-E_N_ (helical content of 40%) in the presence of anionic phospholipid membranes, consistent with the behavior of major antimicrobial peptides [38]. The effect of the peptide–membrane interaction on the phospholipid vesicles stability was assessed by DSC and ^2^H ssNMR analyses. NP-Lun-E_C_ significantly disturbs phospholipid bilayers, lowering transition temperatures (D*T_m_* = 3.3 °C) and enthalpies (D*_trans_H* = 7.3 kJ·mol^−1^) of DMPC:DMPG (3:1) LUVs. On the other hand, the changes in phase transition parameters of the same LUVs were less pronounced in the presence of NP-Lun-E_N_. In addition, whereas NP-Lun-E_N_ had minimal effects on the ^2^H NMR order parameters, NP-Lun-E_C_ demonstrated significant changes in the relative order parameters, further supporting greater membrane perturbation caused by NP-Lun-E_C_. These findings reinforce the relevance of terminal regions as strategic targets for the rational design of bioactive peptides with optimized properties [4,12,36].

Proteolytic resistance was investigated, revealing that the conjugation of lunatin-1 to nanoparticles significantly reduces its degradation rate, delaying the appearance of proteolytic fragments and partially preserving the peptide’s structure. This protective effect provided by the nanoparticles may be attributed to reduced exposure of cleavage-susceptible regions for both NP–peptide conjugates. The enhanced stability observed in the conjugated systems suggests that nanoparticle conjugation is an effective strategy to enhance their therapeutic potential. These findings are consistent with previous studies demonstrating the efficacy of nanoparticle-based delivery systems in protecting bioactive peptides from enzymatic degradation [39,40].

## 4. Materials and Methods

### 4.1. Peptide Synthesis

Lunatin-1 (Lun-1) peptide and its analogs Lun-E_N_ and Lun-E_C_ (Table 1) were synthesized using the Fmoc (9-fluorenylmethyloxycarbonyl) solid-phase synthesis strategy [41]. The syntheses were planned for 300 mg of peptide using the Fmoc Rink^®^ amide resin (0.79 mmol·g^−1^). All steps of peptide synthesis were performed in a polystyrene syringe adapted with a porous polyurethane filter. The coupling steps were performed with 4 equiv. of Fmoc amino acid derivatives activated by treatment with 1,3-diisopropylcarbodiimide (DIC, 4 equiv.) and 1-hydroxybenzotriazole (HOBt, 4 equiv.) in a mixture of N,N-dimethylformamide (DMF):DCM 1:1 (*v*/*v*) during 2 h. Fmoc deprotection was carried out by treatments with 20% 4-methylpiperidine (PIPE) in DMF (2 × 15 min). After each coupling and deprotection step, washes were carried out sequentially with N,N-dimethylformamide (DMF), isopropyl alcohol (IPA), and dichloromethane (DCM).

The cleavage reactions were carried out by adding 5 mL of a TFA:H_2_O:TIS (95:2.5:2.5, *v*:*v*:*v*) solution over 1.0 h. Free peptides were precipitated with cooled 5 mL of diisopropyl ether (Vetec Química Fina—Rio de Janeiro, Brazil), centrifuged (Spinlab^®^ centrifuge, model SL-16RAV-4000, São Paulo, Brazil), and spun down (5 times). The obtained peptides were solubilized in Milli-Q^®^ water for freeze-drying (Terroni LS3000^®^, São Paulo, Brazil).

### 4.2. Preparation of Alumina Nanoparticles and Derivatives Forms

Alumina nanoparticles (NPs)—Step 1

Alumina nanoparticles were synthesized as described by Torres (2018) [20]. Boehmite (AlOOH) was obtained by co-precipitation of aluminum nitrate [Al(NO_3_)_3_, 0.10 mol·L^−1^] and sodium carbonate [Na_2_CO_3_, 0.19 mol·L^−1^], separately solubilized with 150 mL Milli-Q^®^ water at 70 °C under magnetic stirring. The reaction was carried out under reflux for 3.5 h. The resulting gelatinous precipitate was centrifuged at 4500 rpm for 5 min and sequentially washed with water, ethanol, and acetone (10 mL each). The dried material was calcined at 550 °C for 5 h in a muffle furnace to yield alumina nanoparticles (NPs).

Hydroxylation (NP → NP-OH)—Step 2

Hydroxyl groups were introduced by treating 500 mg of NP with 50 mL of hydrogen peroxide 35% (*v*/*v*) under reflux at 100 °C for 15 min. The hydroxylated nanoparticles (NP-OH) were separated by centrifugation (4500 rpm, 5 min) and the obtained solid was washed with water (3 × 10 mL) and ethanol (3 × 10 mL).

Amination of Hydroxylated Alumina (NP-OH → NP-NH_2_)—Step 3

For surface amination, 450 mg of NP-OH were suspended in a APTES/toluene 1:15 (*v*/*v*) solution and refluxed at 110 °C for 2 h. The product (NP-NH_2_) was centrifuged (4500 rpm, 5 min) and sequentially washed with toluene, IPA, DMF, ethanol, and water.

Peptide Conjugation (NP-NH_2_ → Lun-EN or Lun-EC)—Steps 4

Covalent binding of peptides to NP-NH_2_ was performed in ultrapure water (3 mL) using EDAC and HOBt under stirring at 45 °C. First, 45 mg of NP-NH_2_ was suspended in 3 mL of water containing 3.5 mg of EDAC (1 eq.) and 2.8 mg of HOBt (1 eq.). Then, 2 mL of an aqueous solution containing 32.5 mg of free peptide (1 eq.) was added dropwise into a two-neck round-bottom flask over 180 min, until the entire volume had been transferred to the reaction vessel. For quantification purposes for the peptide molecules bound to the alumina nanoparticles (item 4.3), a peptide-containing Fmoc group at the N-terminal residue was used in the NP–peptide conjugation synthesis. The suspension was subsequently centrifuged and washed with water and ethanol (2 × 5 mL each).

After NP–peptide conjugation, the acid-labile N-methyltrityl (Mtt) group of Lys residue was removed using 1.8% (*v*/*v*) TFA in DCM for 3 min at room temperature; this was repeated nine times, using 10 mL solvent per 1 g of resin.

### 4.3. Quantification of Peptide Molecules Bound to the Alumina Nanoparticles

To determine the number of peptide molecules immobilized on the surface of the nanoparticles, the peptides (Lun-E_N_ and Lun-E_C_) were synthesized without performing the Fmoc deprotection step on the last amino acid added. The Fmoc deprotection was carried out only after the peptides were coupled to the nanoparticles. Quantification of peptide molecules immobilized on the nanoparticles was based on the detection of the dibenzofulvene–piperidine adduct formed during the deprotection reaction [20].

Following the coupling, a deprotection reaction was conducted to remove the Fmoc group from the peptides covalently bonded to the nanoparticles. Next, the entire deprotection solution was collected in a volumetric flask to enable the quantification of the dibenzofulvene–piperidine adduct via ultraviolet spectroscopy at 301 nm. The calibration curve (Figure S2) was generated by replicating the Fmoc deprotection process using Rink-Amide resin (0.79 mmol·g^−1^) with a PIPE:DMF solution in a (1:4, *v*:*v*) ratio. This solution was also utilized as a blank during the spectrophotometric analysis.

### 4.4. Morphological and Structural Characterization

#### 4.4.1. Transmission Electron Microscopy

Transmission electron microscopy (TEM) images of the nanoparticles, before and after peptide immobilization, were acquired using a Tecnai G2-20 SuperTwin FEI microscope operating at 200 kV. The samples (NP, NP-Lun-EN, and NP-Lun-EC) were solubilized in ethanol using an ultrasound bath, after which a small drop of each sample was deposited onto a Holey Carbon grid and allowed to dry at room temperature.

#### 4.4.2. Powder X-Ray Diffraction Analysis

Powder X-ray diffraction (XRD) was used to investigate the crystalline structure of the Al_2_O_3_ nanoparticles and their modified forms. The analyses were conducted on a Shimadzu XRD-6000 diffractometer (Kyoto, Japan) using Cu Kα radiation (λ = 1.540560 Å), with the instrument operating at 200 mA and 40 kV. Silicon was used as an external standard.

#### 4.4.3. Zeta Potential and Hydrodynamic Diameter Measurements

Zeta potential and Hydrodynamic Diameter measurements were performed at room temperature (or 25 °C) on a Zetasizer Nano instrument (Malvern^®^ ZS model BI-900, Worcestershire, UK). A 700 µL Malvern^®^ cuvette (model DTS1061) was used for the analysis. The measurements were conducted, utilizing monochromatic light scattering (10 mW Ne laser, λ = 632.4 nm), with the light intensity measured at a 90° angle. Zeta potential (z) were carried out at 0.2 mg·mL^−1^ of NP, NP-OH, NP-NH_2_, NP-Lun-E_N_, and NP-Lun-E_C_ dispersed in a 10 mM Tris-HCl buffer, pH 8.0, containing 100 mM NaCl.

#### 4.4.4. Fourier Transform Infrared Spectroscopy

Infrared absorption spectra were recorded in the range of 4000 to 400 cm^−1^ with 8 cm^−1^ resolution and 32 accumulations. The measurements were performed using a Varian FT-IR spectrophotometer (Agilent, Santa Clara, CA, USA) model 640-IR equipped with an attenuated total reflectance (ATR) accessory Pike Technologies, model GladiATR (Fitchburg, WI, USA) The analysis included solid samples obtained from all stages of nanoparticle synthesis (NP, NP-OH, NP-NH_2_), pure peptides (Lun-E_N_ and Lun-E_C_), and nanoparticles functionalized with the peptides (NP-Lun-E_N_ and NP-Lun-E_C_).

#### 4.4.5. Solid-State Nuclear Magnetic Resonance

Proton-decoupled ^13^C solid-state NMR (ssNMR) spectra were recorded for the free peptides Lun-EN and Lun-EC, as well as for the samples of functionalized nanoparticles, NP, NP-OH, NP-NH_2_, NP-Lun-EN, and NP-Lun-EC. The assays were performed on a Bruker Avance III 500 spectrometer (11.75 T) equipped with a 4 mm CP/MAS probe Bruker BioSpin (Karlsruhe, Germany). Spectral calibration was performed using a standard sample of ^13^C-enriched adamantane as an external reference. Each sample was packed into a zirconium oxide rotor (4 mm diameter) and spun at the magic angle (54.7°) with a rotation speed of 5 kHz. All spectra were collected with a relaxation delay of 1.0 s, a time domain of 27 ms, and a spectral window of 37594 Hz, at 303 K. The cross-polarization (CP) method was used for signal enhancement combined with total sideband suppression (CP-TOSS) [42] in which the CP contact time was set to 4 ms. NMR data were acquired and processed using Bruker TopSpin software (version 3.1). Before Fourier transformation, an exponential apodization function corresponding to a line broadening of 100 Hz was applied to all spectra.

### 4.5. Biological Experiments

#### 4.5.1. Antimicrobial Activity Assay

The peptide Lun-1 and the peptide-conjugated nanoparticles (Lun-E_N_ and Lun-E_C_) were investigated against four bacterial strains—*Pseudomonas aeruginosa*, *Staphylococcus aureus*, *Acinetobacter*, *Escherichia coli*, and *Streptococcus agalactiae*, all from American Type Culture Collection (Manassas, VA, USA). Details can be found in the SM.

#### 4.5.2. Kinetics of Bacterial Death

Bacterial time-kill assays were performed in triplicate, following the methodology of ref. [43] with minor modifications. Staphylococcus aureus was cultured in Mueller–Hinton (MH) broth at an initial concentration of 105 UFC·mL^−1^ and exposed to free peptides and peptide-functionalized nanobiostructures at concentrations corresponding to 0.5×, 1×, 2×, and 4× the MIC (minimum inhibitory concentration). Samples were collected at defined time intervals: 0, 0.5, 1, 2, 4, 6, 8, 10, and 12 h. Cell viability was assessed using the microdrop method described by Romeiro (2001) [44]. Serial dilutions of the bacterial suspensions (10^−1^, 10^−2^, and 10^−3^) were prepared, and 5 µL aliquots from each dilution were plated onto brain–heart infusion (BHI) agar. Plates were incubated for up to 12 h, after which colony counts were performed. The number of colony-forming units (UFC) was calculated by multiplying the colony count by the reciprocal of the dilution factor, and results were expressed as UFC·mL^−1^. This approach provided quantitative data on the time–dependent bactericidal activity of both the free peptides and nanoparticle-conjugated formulations.

### 4.6. Biophysical Experiments

#### 4.6.1. Preparation of Unilamellar Vesicles

For biophysical studies using Circular Dichroism (CD) Spectroscopy, Isothermal Calorimetric Titration (ITC), and Differential Scanning Calorimetry (DSC), large unilamellar vesicles (LUVs) were employed as a membrane-mimetic system. LUVs were prepared with a diameter of 100 nm, using a 3:1 molar ratio of the phospholipids 1-palmitoyl-2-oleyl-sn-glycero-3-phosphocholine (POPC) and 1-palmitoyl-2-oleyl-sn-glycero-3-[phospho-rac-(1-glycerol)] (POPG) in a 10 mM Tris-HCl buffer solution containing 20 mM NaCl at pH 8.5. The preparation followed the dehydration/rehydration of vesicles (DRV) methodology as described by Kirby [45]. Further details are provided in the Supplementary Materials.

#### 4.6.2. Isothermal Calorimetric Titration

ITC analyses were performed in a Malvern ^®^ VP-ITC microcalorimeter (Malvern, UK), at 25 °C, by titration of POPC:POPG (3:1, mol:mol) vesicle solution into the peptide solution. Equipment calibration was performed using Type 1 Milli-Q^®^ deionized water, and the data were processed using Microcal Origin^®^ 7.0 software for ITC analysis. The solutions were pre-degassed using a Malvern^®^ Microcal Thermovac^®^. Experiments began with an initial injection of 130 µL of titrant (LUVs) followed by 29 successive 5 µL injections, with the reaction cell containing 1.4 mL of peptide solution. Injections were performed at 300 s intervals. The experiments consisted of Lun-1, NP-Lun-E_N_, and NP-Lun-E_C_, samples containing 25 µM of peptide concentration, which corresponds to 0.181 mg·ml^−1^ of NP-Lun-E_N_ and 0.174 mg·ml^−1^ of NP-Lun-E_C_. The titrant consisted of 20 mM POPC:POPG (3:1, mol:mol) LUVs suspended in 10 mM Tris-HCl buffer at pH 8.5 containing 100 mM NaCl.

#### 4.6.3. Circular Dichroism Spectroscopy

Circular dichroism spectra were recorded using a JASCO J-815 spectrometer (Easton, MD, USA) equipped with a Peltier Jasco model PTC-423 L temperature control system. Spectra were obtained using a quartz cuvette with a 1 cm optical path at a temperature of 25 °C, covering a wavelength range from 190 to 260 nm with eight consecutive scans per sample. The scan speed was set at 100 nm/min and the response time was 4 s. The bandwidth was 1 nm, and ellipticity readings were taken every 0.2 nm. The Lun-1 (25 µM), NP (0.155 mg·mL^−1^), NP-Lun-E_N_ (0.17 mg·mL^−1^–25 µM of peptide concentration), and NP-Lun-E_C_ (0.18 mg·mL^−1–^25 µM of peptide concentration) samples were (corresponding to 25 µM of peptide in NP-Lun-E_N_ and NP-Lun-E_C_) suspended in 10 mM Tris-HCl buffer solution at pH 8.5 containing 100 mM NaCl or in 200 µM POPC:POPG (3:1, mol:mol) LUVs suspended in the same buffer solution. Data processing and deconvolution calculations to obtain the contents of secondary structures were carried out using the CDpro^®^ program [46].

#### 4.6.4. Differential Scanning Calorimetry

The phase transition profile of 1,2-dimyristoyl-sn-glycero-3-phosphocholine and 1,2-dimyristoyl-sn-glycero-3-phosphocholine/1,2-dimyristoyl-sn-glycero-3-phosphoglycerol (DMPC:DMPG. 3:1, mol:mol) at 3 mM was investigated in the absence and presence of Lun-1, NP, NP-Lun-E_N_, and NP-Lun-E_C_ at different concentrations (Table S1, using a VP-DSC^®^ microcalorimeter (Malvern^®^ Instruments, Malvern, UK). All mixtures of LUVs and peptide–LUVs were prepared immediately prior to the experiments. The samples were pre-degassed and tested against a 10 mM Tris-HCl buffer (pH 7.5) containing 50 mM NaCl in the reference cell. Buffer-only experiments were also performed in all cells for subsequent background subtraction. Each sample underwent three successive heating scans in the temperature range of 10–35 °C at a heating rate of 1.0 °C.min^−1^. Data analyses were performed using Microcal Origin^®^ DSC software version 5.0 (GE HealthCare-Microcal^®^, Amherst, MA, USA), which was employed to subtract the blank and determine the transition temperature (*T_m_*) and transition enthalpy (D_trans_*H*) from the gel phase to the liquid crystalline phase.

#### 4.6.5. ssNMR for Deuterium-Order Parameters Determination in Labeled Phospholipids

Deuterium-order parameters (*S_CD_*) were measured for vesicles composed of POPC and deuterated POPG-*d*_31_ by dissolving the lipids in chloroform (CHCl_3_) 4.2 mg of POPC and 1.5 mg of deuterated POPG (POPG-d31), to reach the mix of POPC:POPG-*d*_31_ at a 3:1 molar ratio. The equivalent amount of peptide or peptide-functionalized nanoparticles was added to achieve a final peptide-to-lipid (P/L) molar ratio of 4%. The solvent was evaporated under a nitrogen stream, followed by vacuum overnight, forming a lipid film on the inner wall of a glass tube (6 mm external diameter).

The lipid film was rehydrated with 26 µL of 10 mM phosphate buffer (pH 7) under vigorous vortexing, followed by sonication in a water bath. Five cycles of cooling (0 °C) and heating (40 °C) were performed thus multilamellar vesicles form. For ^2^H NMR data acquisition, the glass tube containing the sample was directly inserted into the solenoidal coil of a static solid-state NMR (ssNMR) probe.

The ^2^H ssNMR spectra of POPG-*d*_31_ were recorded at 25 °C using a quadrupole pulse echo sequence [47]. The acquisition parameters included a delay of 0.3 s, an echo time of 100 µs, a dwell time of 0.5 µs, and a π/2 pulse of 5 µs. An exponential apodization with a line broadening of 300 Hz was applied before the Fourier transform. The deuterium-order parameters (S_CD_) for the CD_2_ and CD_3_ groups were calculated using the following Equation (1):

(1) $$S_{CD}^{i}=\frac{4}{3}\frac{h}{e^{2}qQ}{\Delta^{i}}_{v}$$

where ${\Delta^{i}}_{v}$ is the quadrupole coupling of segments *i* and (e^2^*qQ*/*h*) is the static C-D quadrupole coupling (167 kHz) [48]. The spectra enable the determination of quadrupole splitting with an accuracy of 1 kHz, corresponding to an error of 0.01 for the order parameters and 0.014 for the relative order parameters. Student’s t can be calculated from the sample average (*D*) and the standard deviation (*S_d_*) of the pair-wise differences between corresponding points of two data sets according to *t = D*/*S_d_*·√*n*, where *n* is the number of data points/differences [49]. From reference t-values, it can be estimated that relative order parameters profiles that are at least 0.02 units apart are significantly different.

### 4.7. Enzymatic Degradation Experiment

Lunatin–AAAE and EAAA–lunatin and their nanoparticle-functionalized counterparts (NP-Lun-E_N_ and NP-Lun-E_C_) were suspended in 20 mM Tris-HCl buffer (pH 8.0) at a final peptide concentration of 100 µM and exposed to enzymatic digestion with trypsin at 37 °C, using an enzyme-to-substrate ratio of 1:50 (*w/w*) in a final volume of 1000 µL. Incubation was performed using an AccuBlock™ Digital Dry Bath (Ciencor),. Aliquots of 200 µL were withdrawn at different time points (1 min, 5 min, 10 min, 30 min, 1 h, 2 h, 3 h, and 4 h), and enzymatic activity was quenched by adding 40 µL of 1 M HCl. Samples were centrifugated and supernatant filtered in whatman^®^ alumina anodisc filter discs 0.02 μm pore size, previously injected in a high-performance liquid chromatography system (Varian^®^ ProStar 315 system equipped with a UV detector), using an Agilent AdvanceBio Peptide Mapping column (4.6 × 150 mm), and 50 µL injection loop.

## 5. Conclusions

Alumina nanoparticles were effectively functionalized with the antimicrobial peptide lunatin. Structural and interaction studies, along with antibacterial and time-kill assays, demonstrated that the C-terminally attached peptide of NP-Lun-E_C_ exhibited superior antimicrobial activity compared to NP-Lun-E_N_, emphasizing the critical role of functionalization site in preserving the antimicrobial properties of the peptide. The results indicate that functionalization at the N-terminus retains the peptide’s structural integrity and activity when bound to the alumina nanostructures. Importantly, the secondary structure of the peptide remained almost unaltered after C-terminal conjugation with nanoparticles. These findings highlight NP-Lun-E_C_ as a promising candidate for the development of peptide-based antimicrobial nanomaterials.

## Figures and Tables

**Table 1:** Primary sequences and acronyms of the peptide and peptide–nanostructures synthetized, highlighting the terminal portion (C-term or N-term) bound to the alumina nanoparticle (NP).

Peptide Acronym	Primary Sequence	Nanostructure Acronym	Peptide Nanostructure Representation
Lun-1	FIGGLLKTLTSFF–NH_2_	-	-
Lun-E_C_	FIGGLLKTLTSFF**AAAE**–NH_2_	NP-Lun-E_C_	Lun-1-AAAE_Cterm_-NP
Lun-E_N_	**EAAA**FIGGLLKTLTSFF–NH_2_	NP-Lun-E_N_	NP-_Nterm_EAAA-Lun-1

**Table 2:** Minimum inhibitory concentration (MIC) values of Lun-1, NP-Lun-E_N_, and NP-Lun-E_C_ against bacterial strains.

Microorganism	MIC (µmol·L^−1^)		
	Lun-1	NP-Lun-E_N_	NP-Lun-E_C_
*Acinetobacter* (*ATCC17978*)	32.1	ND *	40.0
*Escherichia coli* (*ATCC25922*)	64.2	128.5	48.0
*Pseudomonas aeruginosa* (*ATCC27853*)	32.1	ND *	32.1
*Staphylococcus aureus* (*ATCC29213*)	8.0	64.2	16.0
*Streptococcus agalactiae* (*ATCC 29313*)	16.0	128.5	16.0

* ND: not demonstrated antibacterial activity.

**Table 3:** Thermodynamic parameters calculated from the ITC data for Lun-1, NP-Lun-E_N_, and NP-Lun-E_C_.

Thermodynamic Parameters	Samples (50 µM)		
	Lun-1	NP-Lun-E_N_	NP-Lun-E_C_
*n*	7	4	13
*K* (L·mol^−1^)	3.0 × 10^4^ ± 8.0 × 10^2^	5.1 × 10^3^ ± 1.0 × 10^2^	4.1 × 10^4^ ± 3.0 × 10^2^
Δ*G*^0^ (cal·mol^−1^)	−5814	−3478	−7802
Δ*H*^0^ (cal·mol^−1^)	−450 ± 60	−200 ± 30	−650 ± 50
Δ*S*^0^ (cal·mol^−1^.K^−1^)	18	11	24

**Table 4:** Transition temperature (*T_m_*) and transition enthalpy change (Δ*_trans_H*) values for the DMPC:DMPG (3:1, mol:mol) LUVs in the presence of Lun-1, NP-Lun-E_N_, and NP-Lun-E_C_. Estimated uncertainties in triplicate are ± 0.2 °C for *T_m_* and ± 0.5 kJ·mol^−1^ for ∆*_trans_H*.

[Peptide]/µM	*T_m_* (°C)/∆*_trans_H* (kJ·mol^−1^)		
	Lun-1	NP-Lun-E_N_	NP-Lun-E_C_
0	23.1/24.0	23.1/24.0	23.1/24.0
10	22.2/22.1	22.9/24.0	22.7/23.7
25	21.0/18.4	22.9/23.5	22.0/22.0
50	19.3/17.3	22.9/21.5	21.1/19.1
100	18.7/16.2	21.9/17.8	19.8/16.7

## Data Availability

Data are contained within the article and Supplementary Materials.

## Supplementary Materials

The following supporting information can be downloaded at: https://www.mdpi.com/article/10.3390/ph18070952/s1, Figure S1: Mass spectrometry (ESI-MS) profiles of synthetic peptides Lun-E_C_ and Lun-E_N_, confirming molecular weight and structural integrity; Figure S2: X-ray diffractogram of alumina nanoparticles. In black crystal planes and pattern of Rietveld refinement of nanoparticles (NP) of γ-Al_2_O_3_. In red diffractogram for NP-Lun-E_N_ and in blue NP-Lun-E_C_; Figure S3: Mean zeta potential values obtained in triplicate and standard deviation for NP, NP-OH, NP-NH2, NP-Lun-E_N_ and NP-Lun-E_C_ in 10 mM Tris-HCl buffer, 100 mM NaCl, pH 8.5; Figure S4: Average of the hydrodynamic diameter values obtained, in triplicate, and the standard deviation for NP, NP-OH, NP-NH2, NP-Lun-E_N_ and NP-Lun-E_C_ in 10 mM Tris-HCl buffer, 80 mM NaCl, pH 8.5; Figure S5: Curve obtained with the dibenzofulveno-piperidine adduct (product of the deprotection of the resin Rink® amide 0.79 mmol·g^−1^) in PIPE/DMF 1:11.5 (*v*:*v*); Figure S6: Schematic of the functionalization of alumina nanoparticles with the peptides Lun-E_N_ using Route 1, proposed by Torres, 2018; Table S1: Peptide concentration as a Function of NP Concentration (mg.mL^−1^); Table S2: Primary sequence, monoisotopic masses, and *m*/*z* ions for cleavage fragments obtained for free and NP-conjugated peptides.
