# Supplementary material for: Influence of Peptide Conjugation Sites on Lunatin–Alumina Nanoparticles: Implications for Membrane Interaction and Antimicrobial Activity
Source: Pharmaceuticals (Basel). 2025 Jun 24;18(7):952. doi: 10.3390/ph18070952 (PMC12299903; doi:10.3390/ph18070952)
Supplement: Supplementary file 1 [file pharmaceuticals-18-00952-s001.zip › pharmaceuticals-3665855-supplementary.pdf]

Supporting information

Materials

POPC (1-palmitoyl-2-oleoyl-sn-glycero-3-phosphocholine) and POPG (1-palmitoyl-2-oleoyl-sn-glycero-3-[phospho-rac-(1-glycerol)]) were obtained from Avanti Polar Lipids (Alabaster, AL, USA). Amino acids derivatives Fmoc-Phe-OH, Fmoc-Ile-OH, Fmoc-Gly-OH, Fmoc-Leu-OH, Fmoc-Lys(Mtt)-OH, Fmoc-Thr(tBu)-OH, Fmoc-Leu-OH, and Fmoc-Ser(tBu)-OH and Fmoc-Rink amide polystyrene (0,79 mmol.g^{-1}) resin for peptide synthesis were acquired from Novabiochem® (Merck KGaA, Darmstadt, Germany). Ethylene diamine tetraacetic acid (EDTA), copper sulphate pentahydrate ($CuSO_4.5H_2O$), sodium ascorbate ($C_6H_7NaO_6$), acetone, tetrahydrofuran (THF), 3-chloropropyl(triethoxy)silane (CPTES), 3-(4,5-dimethyl-2-thiazolyl)-2,5-diphenyl-2H-tetrazolium bromide (MTT), hydrogen peroxide solution 30% (v:v), chloramphenicol, Dichloromethane (DCM), trifluoroacetic acid (TFA), isopropyl alcohol (IPA), Mueller–Hinton broth and triisopropylsilane were obtained from SigmaAldrich® (Saint Louis, MO). 1,2-diisopropylcarbodiimide (DIC) was acquired from Fluka (Saint Louis, MO). 1-hydroxybenzo-triazole (HOBt), 4-methylpiperidine (PIPE) and 3-triethoxysilylpropan-1-amine (APTES) were acquired from NovaBiochem-Merck (Darmstadt, Germany). N,N-dimethylformamide (DMF) and diisopropyl ether were acquired from Vetec (Duque de Caxias, Brazil). Acetonitrile was acquired from JT Baker (Center Valley, PA). UV/HPLC-grade acetonitrile (ACN) was purchased from Panreac (Barcelona, Spain). Toluene was acquired from Isofar (Rio de Janeiro, Brazil).

Antibacterial Activity Assay

The antibacterial activity of both free peptides and peptide-conjugated alumina nanoparticles was evaluated against four bacterial species: *Pseudomonas aeruginosa, Staphylococcus aureus* (ATCC 29313), *Acinetobacter sp.*, *Streptococcus agalactiae* (ATCC 29313), and *Escherichia coli* (ATCC 25922) (strains from American Type Culture Collection, Manassas, VA, USA). Bacterial culture were grown to the stationary

phase at 35 °C and subsequently transferred to Mueller-Hinton broth according to the guidelines established by the M07-A9 CLSI. Methods for the dilution antimicrobial susceptibility tests for bacteria that grow aerobically were from the Approved Standard—Ninth Edition. CLSI document M07-A9. Wayne, PA: Clinical and Laboratory Standards Institute; 2012. Bacterial suspensions were adjusted to the turbidity equivalent to 0.5 McFarland standard (approximately 5 × 10^5 CFU·mL^{-1}), by spectrophotometric measurement at 625 nm (Bel Photonics S2000 UV, São Paulo, Brazil). The suspension was then diluted 1:20, and 50 μL aliquots were distributed into 96-well flat-bottom microplates (Nümbrecht, Germany).

Chloramphenicol (30 μg·mL^{-1}; 92.8 μM) was used as standard drug and served as positive control for growth bacterial inhibition. Wells containing 50 μL of bacterial inoculum and 50 μL of sterile culture medium were used as negative controls. Free peptides were tested at a maximum concentration of 128.5 μM and diluted via serial dilution (1:2) up to seven times in Mueller–Hinton broth. For the nanoparticle–peptide conjugations concentrations were adjusted according to the degree of functionalization to ensure accurate dosing of the active peptide component.

Plates were incubated at 37 °C for 20 hours. After incubation, 30 μL of a 0.01% (w/v) resazurin solution (10-oxido-7-oxophenoxazin-10-ium-3-olate; Sigma-Aldrich, St. Louis, MO, USA) was added to each well. Plates were protected from light and incubated for an additional 4 hours to allow colorimetric development. The tests whose bacteria did not develop a pink color after incubation (maintaining the blue coloration) were considered to possess antibacterial activity. The experiments were performed in triplicate

Preparation of Unilamellar Vesicles

Large unilamellar vesicles (LUVs) were prepared using 1-palmitoyl-2-oleoyl-sn-glycero-3-phosphocholine (POPC) and 1-palmitoyl-2-oleoyl-sn-glycero-3-[phospho-rac-(1-glycerol)] (POPG) in a molar ratio of 3:1. Lipids were dissolved in 1 mL of chloroform to yield a 20 mM solution. The preparation followed the dehydration–rehydration vesicle (DRV) method described by Kirby and Gregoriadis (1984). The lipid solution was

transferred to a glass test tube, and the chloroform was evaporated under a nitrogen gas stream while rotating the tube to ensure the uniform formation of a thin lipid film along the inner wall. The lipid film was then hydrated with Tris-HCl buffer (10 mM Tris-HCl, 20 mM NaCl, pH 8.5), resulting in the formation of multilamellar vesicles.

To obtain unilamellar vesicles, the suspension underwent eight cycles of freezing in liquid nitrogen (−196 °C), thawing in a 40 °C water bath, and subsequent sonication for 2 minutes. This process yielded unilamellar vesicles with variable sizes, which were submitted to extrusion (eight times) through two 100 nm polycarbonate membranes in an Avanti mini-extruder (Avanti®, Polar Lipids, Inc.,Alabaster, AL).

Supplementary Figures

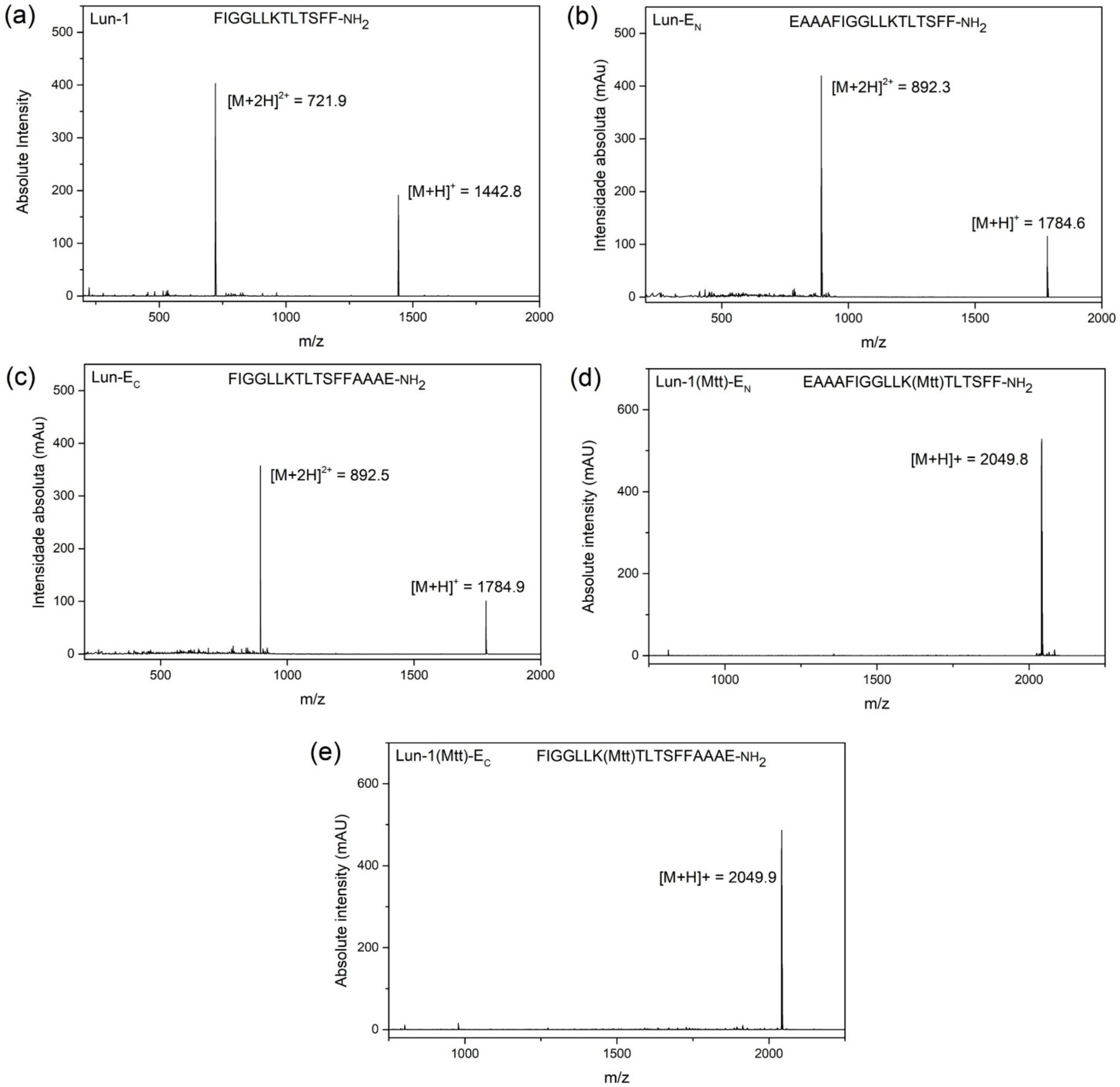

Figure S1. Mass spectrometry (ESI-MS) profiles of synthetic peptides Lun-1 (a), Lun-E_N (b), and Lun-E_C (c), and mass spectrometry (MALDI-ToF) of N-methyltrityl (Mtt) Lys protect derivatives Lun(Mtt)-E_N (d) and Lun(Mtt)-E_C (e).

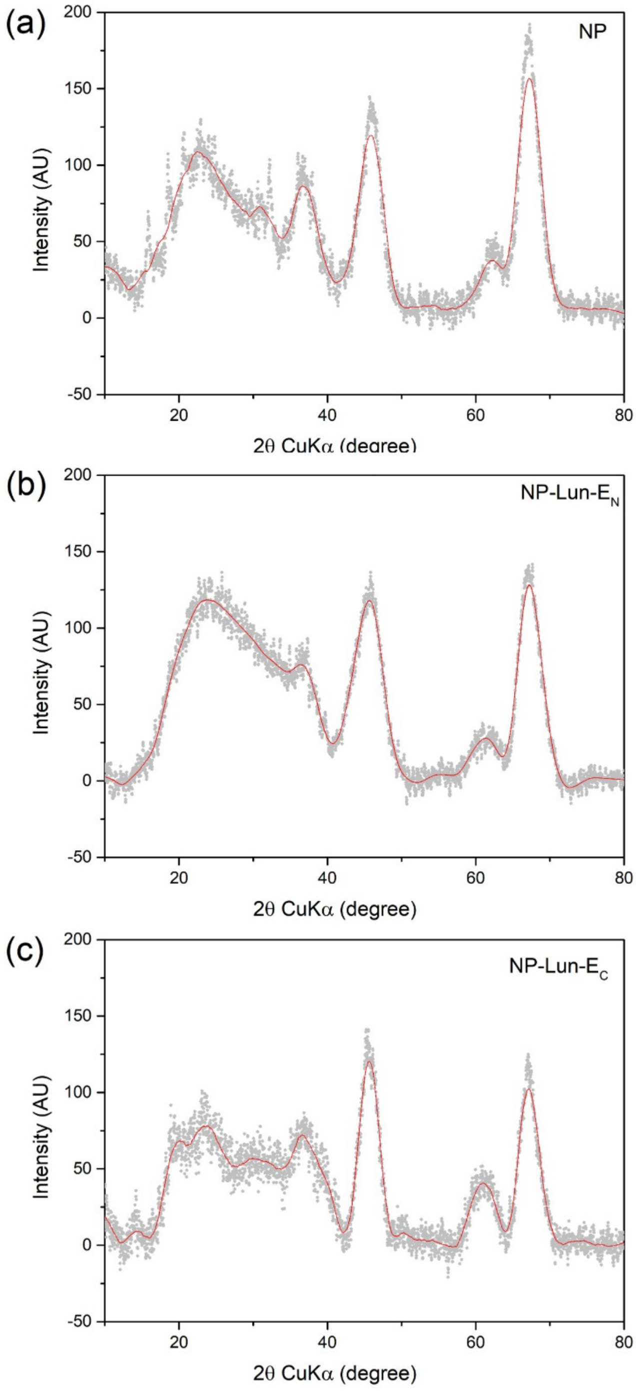

Figure S2. X-ray diffraction pattern of the Al_2O_3 nanoparticles and derivative NP–peptide forms.

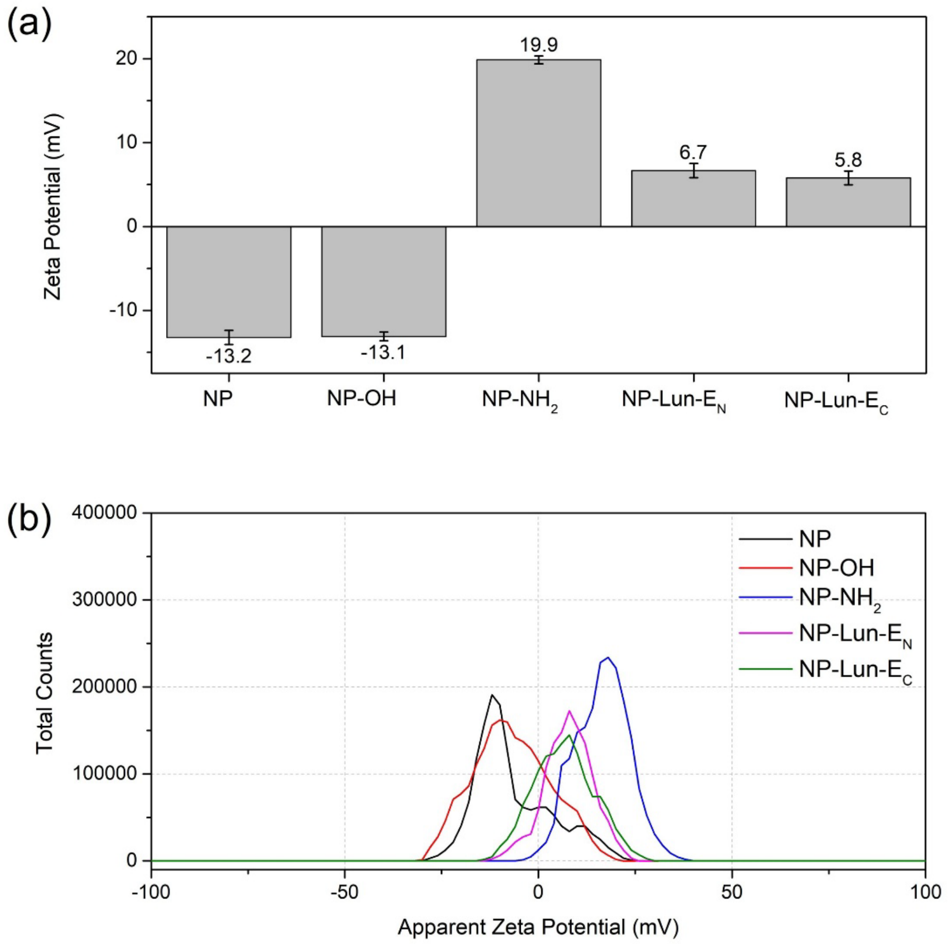

Figure S3. Mean zeta potential (a) and apparent zeta potential distribution (b) of NP, NP-OH, NP-NH_2, and NP–peptides suspended in 10 mmol.L^{-1} Tris-HCl buffer solution, pH 8.0, containing 100 mM NaCl.

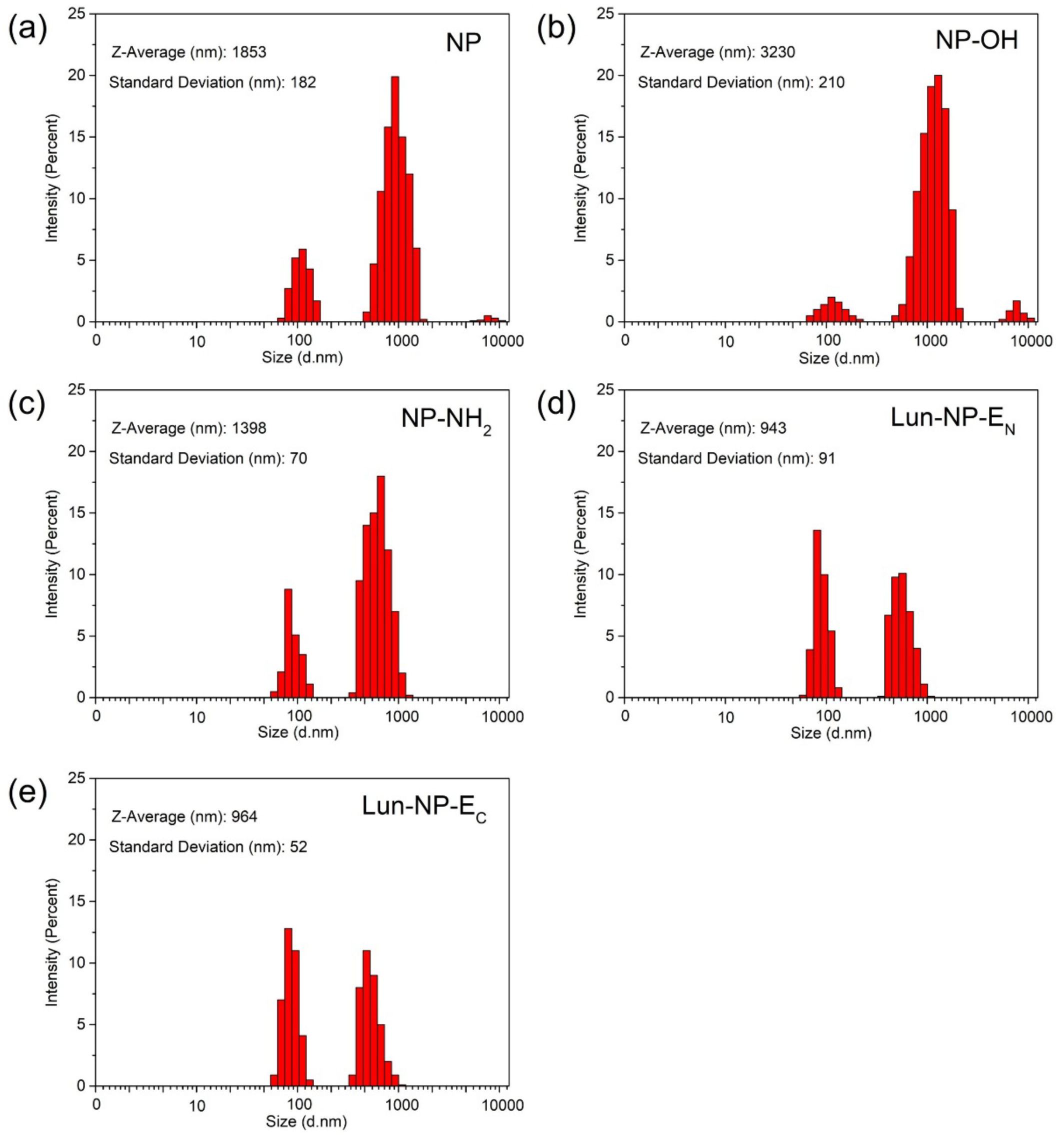

Figure S4. Size distribution (nm) obtained for NP, NP-OH, NP-NH_2, NP-Lun-E_N, and NP-Lun-E_C suspended in 10 mM Tris-HCl buffer, 100 mM NaCl, pH 8.0.

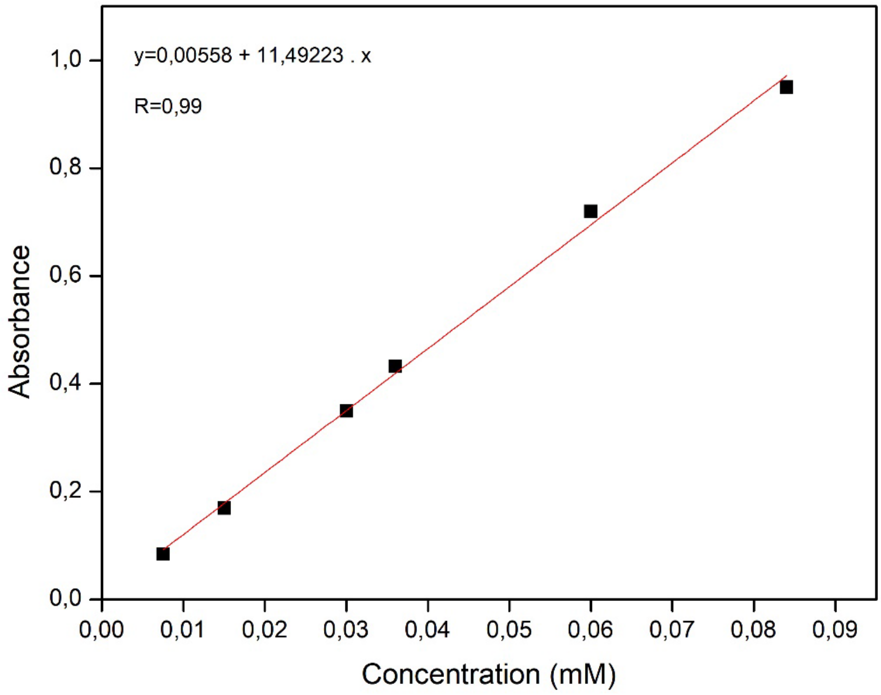

Figure S5. Curve obtained with the dibenzofulveno–piperidine adduct (product of the deprotection of the resin Rink® amide 0.79 mmol·g^{-1}) in PIPE/DMF 1:11.5 (v:v).

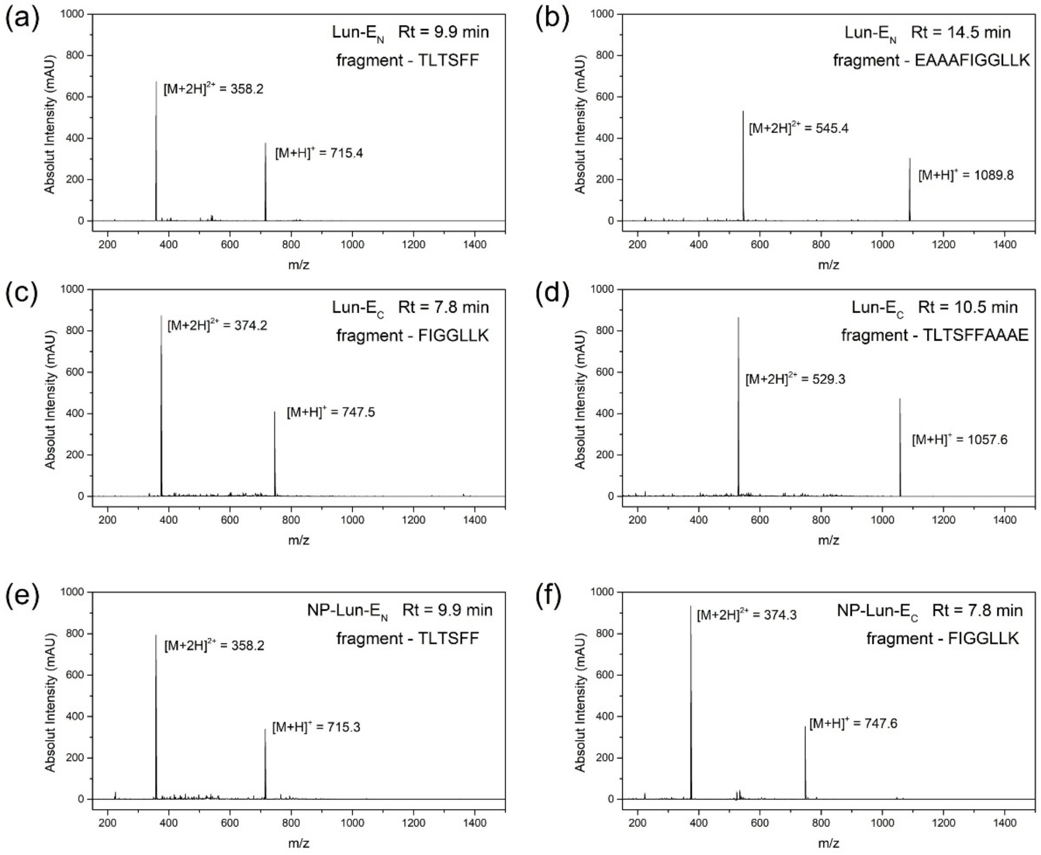

Figure S6. Mass spectrometry (ESI-MS) profiles of for cleavage fragments obtained for free and NP-conjugated peptides

Supplementary Table

Table S1. Peptide concentration as a function of NP concentration (mg·mL^{-1})

Nanobiostructure	Concentration of peptide (µM)	Concentration of alumina nanoparticle (mg·ml^{-1})
NP-Lun-E_N	0	0
NP-Lun-E_N	10	0.07
NP-Lun-E_N	25	0.17
NP-Lun-E_N	50	0.35
NP-Lun-E_N	100	0.67
NP-Lun-E_C	0	0.00
NP-Lun-E_C	10	0.07
NP-Lun-E_C	25	0.18
NP-Lun-E_C	50	0.36
NP-Lun-E_C	100	0.72

Table S2. Primary sequence, monoisotopic masses, and m/z ions for cleavage fragments obtained for free and NP-conjugated peptides

Peptide	Rt (min)	Fragment	Monoisotopic Mass (Da)	$[M + H]^+$ (m/z)	$[M + 2H]^{2+}$ (m/z)
Lun-E_N	9.9	TLTSFF	714.3	715.4	358.2
	14.5	EAAAFIGGLLK	1088.6	1089.8	545.4
Lun-E_C	7.8	FIGGLLK	746.4	747.5	374.2
	10.5	TLTSFFAAAE	1056.5	1057.6	529.3
NP-Lun-E_N	9.9	TLTSFF	714.3	715.3	358.2
NP-Lun-E_C	7.8	FIGGLLK	746.4	747.6	374.3
